# Robust immunity to influenza vaccination in haematopoietic stem cell transplant recipients following reconstitution of humoral and adaptive immunity

**DOI:** 10.1002/cti2.1456

**Published:** 2023-06-27

**Authors:** Wuji Zhang, Louise C Rowntree, Ramona Muttucumaru, Timon Damelang, Malet Aban, Aeron C Hurt, Maria Auladell, Robyn Esterbauer, Bruce Wines, Mark Hogarth, Stephen J Turner, Adam K Wheatley, Stephen J Kent, Sushrut Patil, Sharon Avery, Orla Morrissey, Amy W Chung, Marios Koutsakos, Thi HO Nguyen, Allen C Cheng, Tom C Kotsimbos, Katherine Kedzierska

**Affiliations:** ^1^ Department of Microbiology and Immunology University of Melbourne, at the Peter Doherty Institute for Infection and Immunity Melbourne VIC Australia; ^2^ Department of Infectious Diseases Alfred Health Melbourne VIC Australia; ^3^ World Health Organisation (WHO) Collaborating Centre for Reference and Research on Influenza, at the Peter Doherty Institute for Infection and Immunity Melbourne VIC Australia; ^4^ Product Development Medical Affairs, Infectious Diseases F. Hoffmann-La Roche Ltd Basel Switzerland; ^5^ Burnet Institute Melbourne VIC Australia; ^6^ Infection and Immunity Program, Monash Biomedicine Discovery Institute, and Department of Microbiology Monash University Clayton VIC Australia; ^7^ Melbourne Sexual Health Centre, Infectious Diseases Department, Alfred Health, Central Clinical School Monash University Melbourne VIC Australia; ^8^ Malignant Haematology and Stem Cell Transplantation Service, Department of Clinical Haematology The Alfred Hospital Melbourne VIC Australia; ^9^ School of Public Health and Preventive Medicine Monash University Clayton VIC Australia; ^10^ Infection Prevention and Healthcare Epidemiology Unit Alfred Health Melbourne VIC Australia; ^11^ Department of Respiratory Medicine The Alfred Hospital Melbourne VIC Australia; ^12^ Department of Medicine, Central Clinical School, The Alfred Hospital Monash University Melbourne VIC Australia; ^13^ Global Station for Zoonosis Control, Global Institution for Collaborative Research and Education (GI‐CoRE) Hokkaido University Sapporo Japan

**Keywords:** antibodies, antibody landscapes, B cells, haematopoietic stem cell transplant recipients, influenza vaccination, system serology

## Abstract

**Objectives:**

Influenza causes significant morbidity and mortality, especially in high‐risk populations. Although current vaccination regimens are the best method to combat annual influenza disease, vaccine efficacy can be low in high‐risk groups, such as haematopoietic stem cell transplant (HSCT) recipients.

**Methods:**

We comprehensively assessed humoral immunity, antibody landscapes, systems serology and influenza‐specific B‐cell responses, together with their phenotypes and isotypes, to the inactivated influenza vaccine (IIV) in HSCT recipients in comparison to healthy controls.

**Results:**

Inactivated influenza vaccine significantly increased haemagglutination inhibition (HAI) titres in HSCT recipients, similar to healthy controls. Systems serology revealed increased IgG1 and IgG3 antibody levels towards the haemagglutinin (HA) head, but not to neuraminidase, nucleoprotein or HA stem. IIV also increased frequencies of total, IgG class‐switched and CD21^lo^CD27^+^ influenza‐specific B cells, determined by HA probes and flow cytometry. Strikingly, 40% of HSCT recipients had markedly higher antibody responses towards A/H3N2 vaccine strain than healthy controls and showed cross‐reactivity to antigenically drifted A/H3N2 strains by antibody landscape analysis. These superior humoral responses were associated with a greater time interval after HSCT, while multivariant analyses revealed the importance of pre‐existing immune memory. Conversely, in HSCT recipients who did not respond to the first dose, the second IIV dose did not greatly improve their humoral response, although 50% of second‐dose patients reached a seroprotective HAI titre for at least one of vaccine strains.

**Conclusions:**

Our study demonstrates efficient, although time‐dependent, immune responses to IIV in HSCT recipients, and provides insights into influenza vaccination strategies targeted to immunocompromised high‐risk groups.

## Graphical abstract

In this study, we found inactivated influenza vaccine (IIV) significantly increased hemagglutination inhibition (HAI) titres, hemagglutinin (HA)‐specific B cells and HA head‐specific IgG1 and IgG3 antibodies in haematopoietic stem cell transplant (HSCT) recipients, similar to healthy controls. Higher HAI titres with broader cross‐reactivity were observed in HSCT recipients with greater time‐interval after HSCT. Two doses of IIV only boosted the humoral responses marginally in low responders.

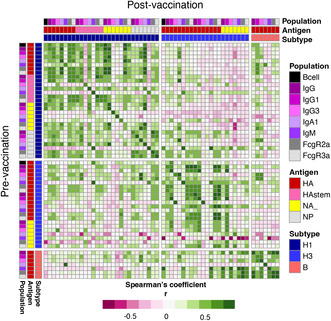

## Introduction

Seasonal influenza virus infections cause significant morbidity and mortality, resulting in ~500 000 deaths worldwide annually in pre‐COVID‐19 pandemic years.^1^ While fewer influenza virus infection cases were reported during the COVID‐19 pandemic, the infection rates are now on the increase again due to the relaxation of public health measures.^2^ For a long time, two influenza A virus (IAV) subtypes, A/H1N1 and A/H3N2, and two influenza B virus (IBV) lineages, B/Yamagata and B/Victoria, co‐circulated in the human population.^3^ Since 2016, all four subtype strains have been included in the inactivated influenza vaccine (IIV) available to Australia. Although influenza virus infection is self‐resolving and mostly causes mild disease in healthy adults, severe prolonged disease can occur in high‐risk groups such as children, the elderly, pregnant women and immunocompromised individuals including haematopoietic stem cell transplant (HSCT) recipients.^4^ Reconstitution of the immune system following HSCT depends on age of recipients, intensity of the conditioning regimen and complicating factors. Generally, the number of innate immune cells and their function are reconstituted within 2 months after transplantation, but the full reconstitution of adaptive T‐ and B‐cell numbers and their function can take years.^5^ In addition, complications such as graft versus host disease and treatment with immunosuppressive drugs can further impair recipients' immunity.^6^, ^7^ As a result, HSCT recipients are at higher risk of severe influenza disease, leading to more prolonged viral shedding, higher hospitalisation rates, severe complications, including lower respiratory tract infections (LRTIs) and higher mortality rates than the healthy population.^8^, ^9^, ^10^, ^11^, ^12^, ^13^, ^14^ Influenza vaccination is thus highly recommended for HSCT recipients more than 6 months after transplantation.^15^

Annual influenza vaccination remains the most effective way of preventing influenza virus infections. However, vaccine responses in HSCT recipients can be sub‐optimal, especially in the first 6 months' post‐transplant, likely due to a lack of immune reconstitution and/or strong immunosuppressive regimens.^16^ Additionally, HSCT recipients' immune responses towards IIV can still be impaired at later stages, compared to healthy individuals. While the standard IIV can induce sub‐optimal humoral and CD4^+^ T‐cell responses in HSCT recipients, IIV can provide some level of protection as vaccinated HSCT recipients have lower rates of influenza virus infection and LRTIs than non‐vaccinated HSCT recipients.^17^, ^18^, ^19^ Adjuvanted and high‐dose vaccines have been trialled to improve immune responses in HSCT recipients. However, these led to either no improvement in serological responses when compared with patients receiving standard IIV, or higher immunogenicity was accompanied with adverse responses, such as inject‐site reactions.^16^, ^20^ Studies focusing on administering two doses of influenza vaccine found that using the AS03‐adjuvanted A/H1N1pdm09 vaccine increased serological response after the second dose, while the effects of two unadjuvanted IIV doses on immune responses in HSCT patients are still unclear,^21^, ^22^, ^23^, ^24^, ^25^ with one recent study showing no differences in B‐cell and CD4^+^ T‐cell responses.^25^ Given the conflicting evidence, more comprehensive analyses are needed to investigate immunity induced for optimal influenza vaccination regimens for HSCT patients.

In our study, we assessed immune responses to IIV at cellular and humoral levels in a cohort of HSCT recipients at least 1 year post transplantation. We found that a subgroup of HSCT recipients had particularly robust and broad antibody responses towards A/H3N2 strains, including an unencountered future strain. Using a multiplex antigen array and systems serology approach, we found that HSCT recipients with higher IIV responses were at a late‐stage post‐transplantation, and this correlated with improved immune reconstitution of influenza‐specific humoral immunity. Conversely, some HSCT patients displayed minimal antibody and B‐cell responses, and this was linked to shorter time intervals after HSCT. Thus, our study suggests that antibody and influenza‐specific B‐cell responses in HSCT recipients are greatly affected by the time post‐transplantation. Administration of a second dose of IIV did not greatly improve influenza‐specific B‐cell or antibody responses in HSCT recipients. The findings of our study have implications for the design of effective vaccination strategies in groups at high risk of severe influenza disease.

## Results

### Influenza vaccination cohort

**Figure 1:**
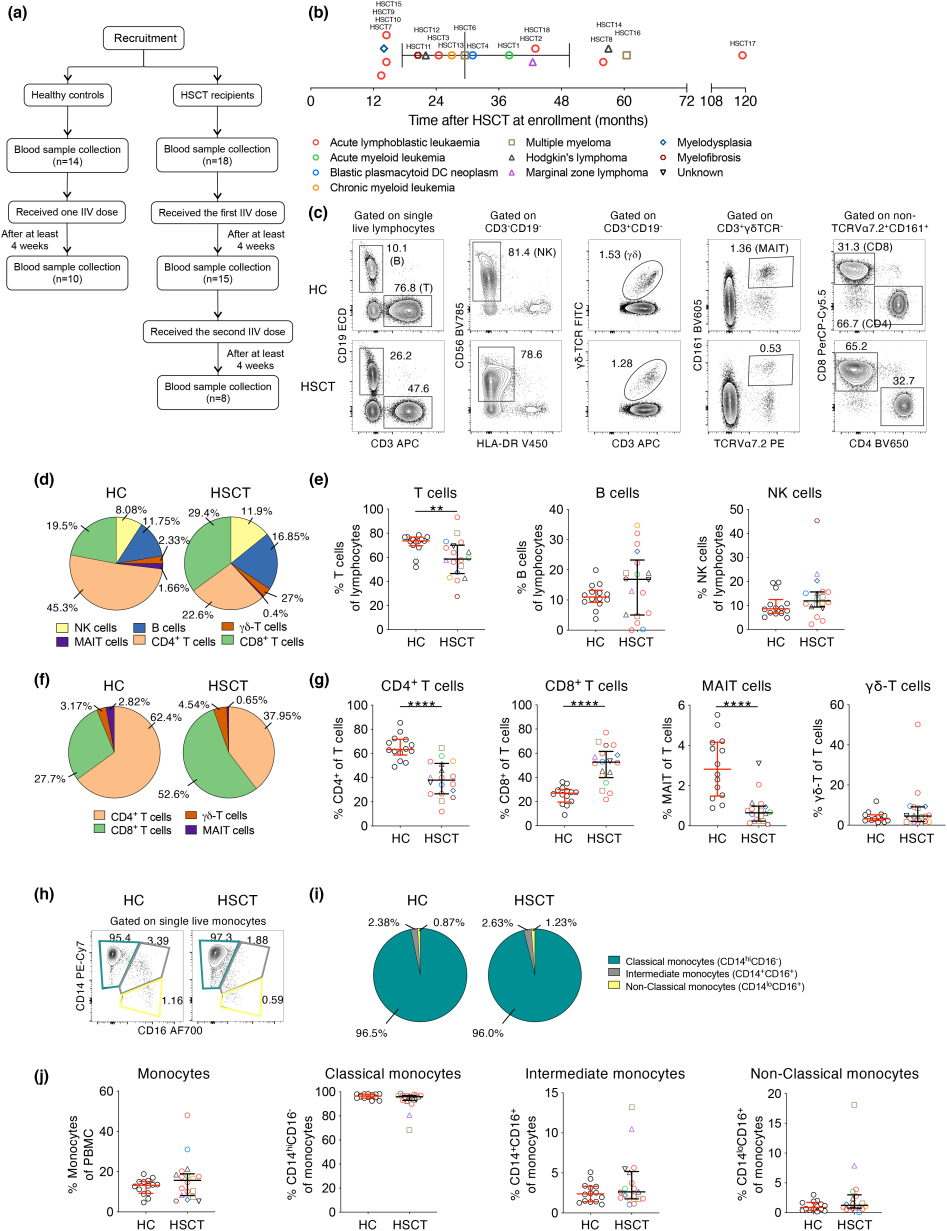
Baseline cellular PBMC composition in HSCT recipients. **(a)** Recruitment of participants and study design. **(b)** Underlying disease and time after transplantation at enrolment of HSCT recipients. **(c)** Gating strategy of PBMC populations. **(d)** Distribution and **(e)** frequency of PBMC subsets. **(f)** Distribution and **(g)** frequency of T‐cell subsets. **(h)** Representative graphs, **(i)** distribution and **(j)** frequency of monocytes and subsets. Pie charts indicate median frequency. Bars indicate median and interquartile range. Symbols of HSCT recipients were the same as in **(b)**. Technical replicates were not performed due to limited patient samples. Significance between the two groups was determined using the Mann–Whitney *U*–test (n_HC_ = 14, n_HSCT_ = 18; **P < 0.01, ***P < 0.001, ****P < 0.0001).

To study immune responses towards IIV, 18 HSCT recipients and 14 age‐matched healthy controls (HC) were recruited to the BMT‐V cohort (age range 18–65 years; median age of 33 (HC) and 35 (HSCT) (Supplementary table 1)). All HSCT and HC participants were bled at baseline and received the IIV (2015 Fluvax®), which was composed of 15 μg of HA from A/California/07/2009 (H1N1) pdm09–like virus, A/Switzerland/9715293/2013 (H3N2)–like virus and B/Phuket/3073/2013–like virus (Yamagata lineage). Fifteen HSCT and 10 HC participants provided a follow‐up sample at least 4 weeks after one vaccination dose (Figure 1a). HSCT participants received a second IIV dose, but only eight of them were blood sampled at least 4 weeks after the second IIV dose. HSCT recipients received IIV at least 12 months after transplantation, with a median of 29.5 months (Figure 1b and Supplementary table 2).

### Altered cellular composition and differentiation state following immune reconstitution in HSCT recipients

We first assessed the immunological status of HSCT recipients prior to vaccination. Cytokine profiles were comparable between HSCT and HC, with only minimal levels being detectable in both groups (Supplementary figure 1a and b). However, the composition of PBMCs exhibited marked differences with HSCT recipients having significantly lower T‐cell frequencies (median 0.8‐fold) than healthy controls (median HSCT 58.6% and HC 73.6%, *P* = 0.0052; Figure 1c–e and Supplementary figure 2), but B‐cell and NK‐cell frequencies were similar. HSCT recipients also had lower CD4^+^ T‐cell and MAIT‐cell frequencies of total T cells (median 0.6‐ and 0.23‐fold respectively), while CD8^+^ T‐cell frequencies were 1.9‐fold higher than those in healthy controls (Figure 1f and g). No differences were observed in the frequency of monocytes, or their subsets based on the surface expression of CD14 and CD16 (Figure 1h–j).

**Figure 2:**
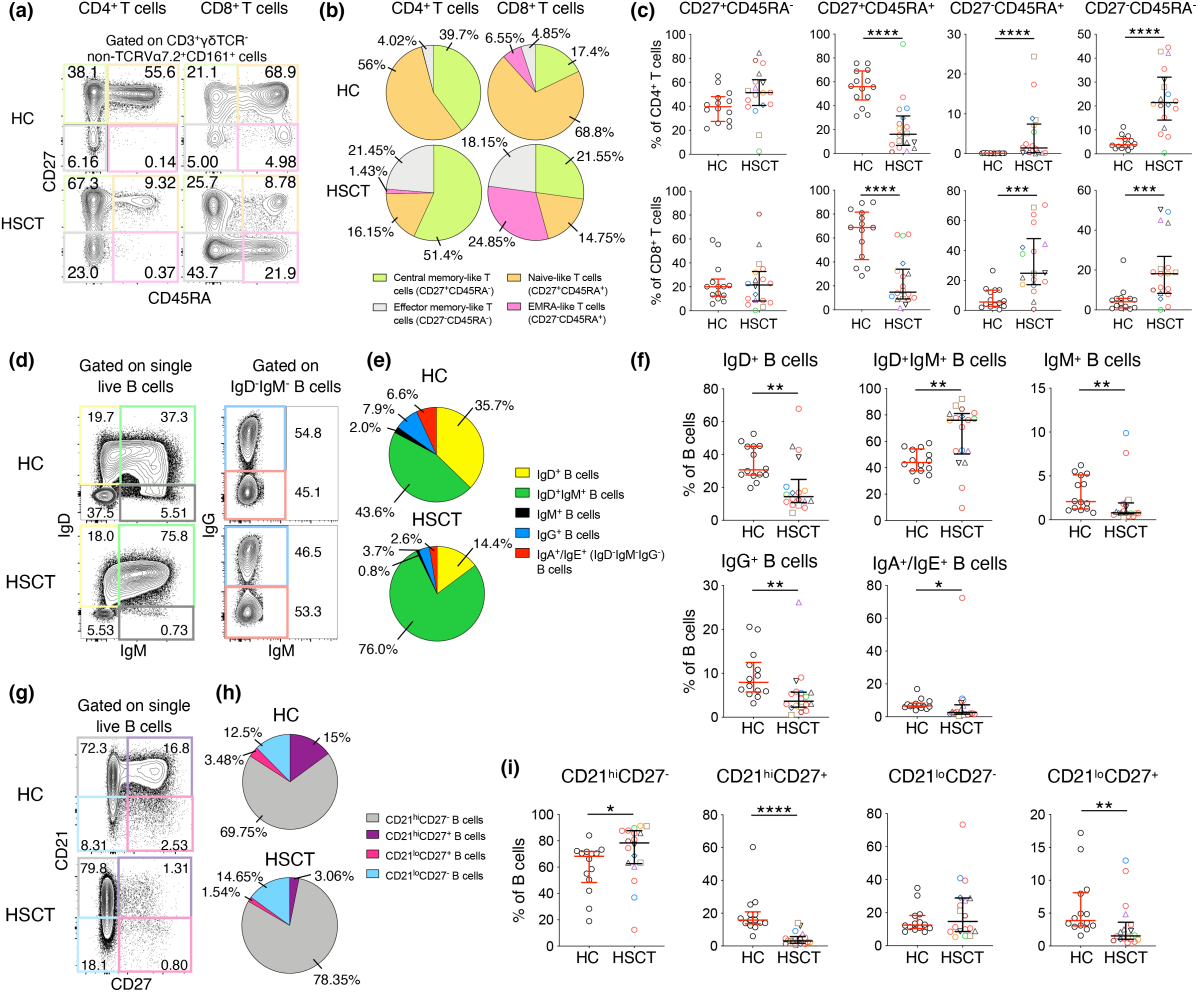
HSCT recipients have higher frequency of CD21^hi^CD27^−^ B cells. **(a)** Representative graphs, **(b)** distribution and **(c)** frequency of CD4^+^ and CD8^+^ T‐cell phenotypes. **(d)** Representative graphs, **(e)** distribution and **(f)** frequency of B‐cell isotypes. **(g)** Representative graphs, **(h)** distribution and **(i)** frequency of B‐cell phenotypes. Pie charts indicate median frequency. Bars indicate median and interquartile range. Symbols of HSCT recipients were the same as in Figure 1b. Technical replicates were not performed due to limited patient samples. Significance between the two groups was determined using the Mann–Whitney *U*‐test (n_HC_ = 14, n_HSCT_ = 18; *P < 0.05, **P < 0.01, ****P < 0.0001).

To further assess the reconstitution of adaptive immunity by their cell differentiation states, CD4^+^ and CD8^+^ T cells were grouped into T‐cell naïve‐like (TN; CD27^+^CD45RA^+^), central memory‐like (TCM; CD27^+^CD45RA^−^), effector memory‐like (TEM; CD27^−^CD45RA^−^) and effector memory CD45RA^+^‐like (TEMRA; CD27^−^CD45RA^+^) populations (Figure 2a). HSCT recipients displayed substantially lower frequency of TN‐like cells for both CD4^+^ (median HSCT 16.2% and HC 56.0%, *P* < 0.0001) and CD8^+^ (median HSCT 14.8% and HC 68.8%, *P* < 0.0001) T cells (Figure 2b and c). Accordingly, higher frequencies of TEM‐ and TEMRA‐like populations and a trend for TCM‐like subset were observed in HSCT (all *P* < 0.001), likely reflecting the homeostatic proliferation of memory T‐cell populations that survived depletion regimens, consistent with previous reports.^26^

In contrast to T cells, HSCT recipients showed levels of unswitched IgD^+^IgM^+^ B cells higher than those of the HC group (median 76.0% for HSCT and 44.0% for HC; *P* = 0.0054), while the frequency of IgD^+^IgM^−^ and class‐switched IgM^+^, IgG^+^ and IgA^+^/IgE^+^ (based on IgD^−^IgM^−^IgG^−^ population) B cells were lower in HSCT recipients (all *P* < 0.05, Figure 2d–f; Supplementary figure 3a). Consistent with the higher frequency of isotype‐unswitched B cells, when analysing B‐cell phenotypes based on memory marker CD27 and Complement Receptor type 2 (CR2/CD21), HSCT recipients had a higher frequency of CD21^hi^CD27^−^ B cells (median 78.4% for HSCT and 68.2% for HC; *P* = 0.0399) and lower frequencies of CD21^hi^CD27^+^ and CD21^lo^CD27^+^ B cells (both *P* < 0.01, Figure 2g–i), unlike the T‐cell compartment.

Overall, cellular compartments within HSCT recipients at least 12 months after transplantation vary in their differentiation state compared to healthy controls, having increased memory T‐cell populations, but contrastingly more isotype‐unswitched and CD21^hi^CD27^−^ B cells in peripheral blood.

### HSCT recipients mount robust humoral immune responses following IIV

**Figure 3:**
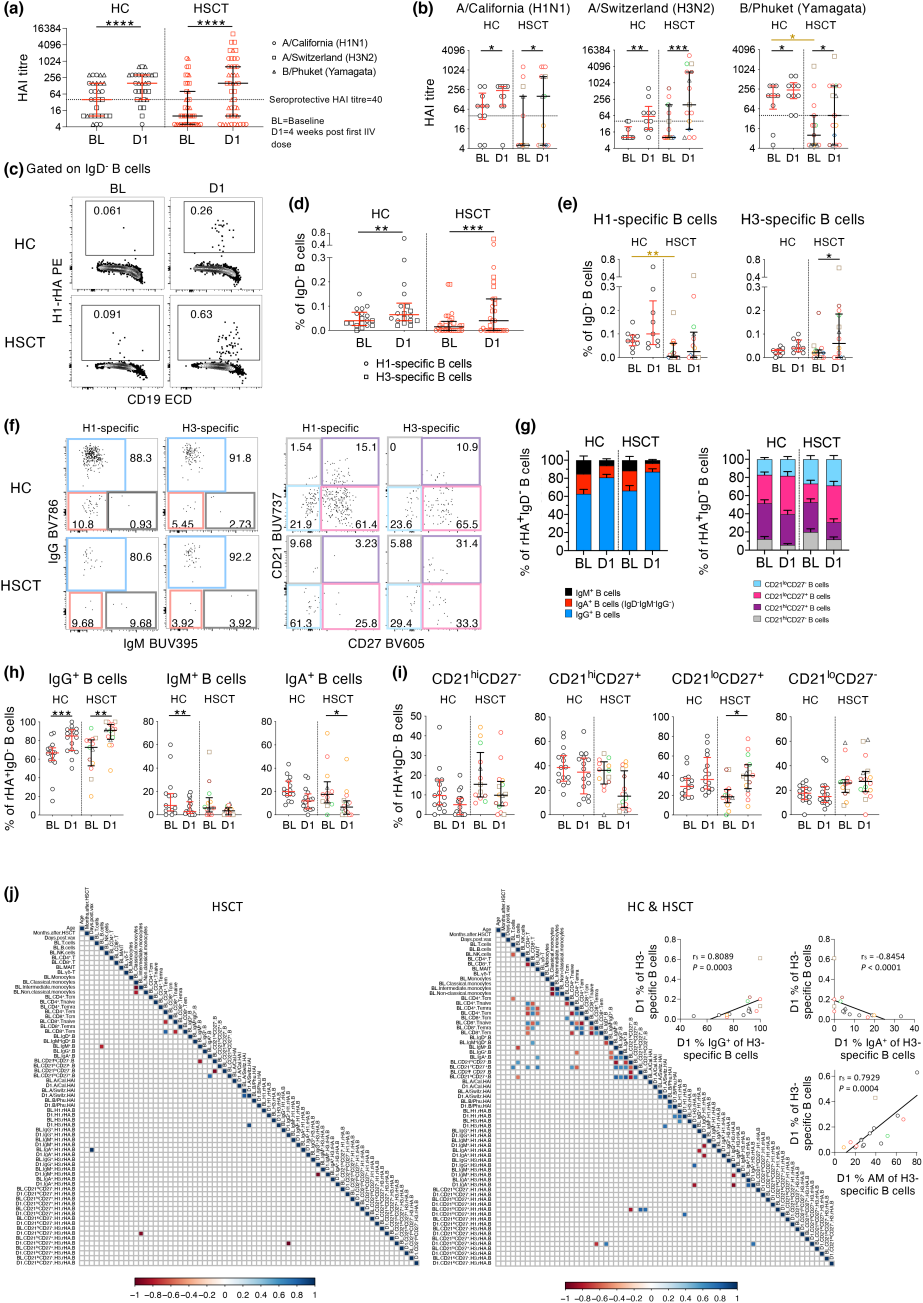
Humoral responses in HSCT recipients following one dose of IIV. **(a)** Pooled or **(b)** separate analyses of HAI titre against vaccine strains (n_HC_ = 10, n_HSCT_ = 15). **(c)** Representative graphs of recombinant HA‐specific (rHA) B cells. **(d)** Pooled or **(e)** separate analyses of influenza‐specific B‐cell frequency. Only donors with detectable H1/H3 influenza‐specific cells both at baseline and after one dose were included (n_HC_ = 9, n_HSCT_ = 14). **(f)** Representative graphs, **(g)** distribution and frequency of influenza‐specific B cell **(h)** isotypes and **(i)** phenotypes. Samples with at least 10 rHA‐specific B cells were further analysed for isotype and phenotype (n_HC_ = 9, H1: n_HSCT_ = 7, H3: n_HSCT_ = 9). **(j)** Correlations between clinical and immunological features for HSCT alone (left) and HC & HSCT (right). Bars indicate median and interquartile range. Stacked bar graphs indicate mean (+ SEM) frequency. Horizontal dotted lines indicate seroprotective HAI titre at 40. Symbols of HSCT recipients were the same as in Figure 1b. Technical replicates were not performed due to limited patient samples. Significance between baseline and after one dose was determined using the Wilcoxon test. Significance between different groups was determined using the Mann–Whitney *U*–test (shown in brown). Correlation was determined with Spearman's correlation. *P < 0.05, **P < 0.01, ***P < 0.001, ****P < 0.0001.

Having characterised baseline immune profiles of HSCT recipients compared to healthy controls, we determined humoral responses to IIV at the serological and cellular level using haemagglutination inhibition (HAI) assay and recombinant HA (rHA) B‐cell profiling. Both groups had a significant increase in HAI titre after the first dose of IIV when data from three vaccine strains were analysed together or separately (Figure 3a and b). Influenza‐specific B‐cell response was determined using rHA probes conjugated to streptavidin‐fluorochrome complexes.^27^, ^28^ Specificity of these rHA probes has been extensively validated by various methods in several studies.^27^, ^28^, ^29^, ^30^ Probes matching the two IAV vaccine strains were used, A/California/2009‐H1 and A/Switzerland/2013‐H3, to detect H1‐ and H3‐specific memory B cells, respectively, within the IgD^−^ B‐cell population. Similar to HAI responses, both HC and HSCT groups had higher frequencies of H1‐ or H3‐specific B cells after vaccination when data from the two strains were pooled (Figure 3c and d; Supplementary figure 3b), although only H3‐specific B cells in HSCT recipients increased significantly when analysed separately (median 0.02% (BL) to 0.06% (D1), *P* = 0.0117, Figure 3e). Interestingly, however, the HSCT group had lower baseline levels of B/Phuket HAI titres and H1‐specific B cells than those of the HC group (*P* = 0.0190 and *P* = 0.0037 respectively; Figure 3b and e), indicating suboptimal reconstitution of influenza‐specific immunity.

To determine the isotype distribution and phenotype of H1‐ and H3‐specific B cells, we analysed HSCT recipients (datapoints *n* = 14) that had detectable levels of influenza‐specific B cells using pooled H1 and H3 B‐cell probe data. Influenza‐specific B cells were predominantly IgG^+^ in both groups at baseline (mean 67.0% for HC and 72.2% for HSCT), with frequencies increasing significantly after vaccination (both *P* < 0.01, Figure 3f–h). Influenza‐specific B cells were less of IgM^+^ in HC and less of IgA^+^ in HSCT recipients following IIV. Influenza‐specific B cells were mainly of CD21^hi^CD27^+^ at baseline (mean 52.4% for HC and 58.6% for HSCT). CD21^lo^CD27^+^ B‐cell probe frequency increased in the HSCT group (median BL 17.6% to D1 40.1%, *P* = 0.0134), and a similar trend was observed in the HC group (median BL 21.6% to D1 36.2%), albeit not significant (Figure 3f, g and i).

To investigate the correlates of better baseline immune system recovery and post‐vaccine immune responses, we correlated age, months after HSCT and days post vaccination with their immunological variants. However, all these three factors did not correlate with immunological factors, when looking at HSCT alone or combing HC and HSCT participants (Figure 3j). Interestingly, % of H3 rHA‐specific B cells at D1 correlated with % IgG^+^ while reversely correlated with % IgA^+^ H3 rHA‐specific B cells. Similarly, % CD21^lo^CD27^+^ H1 rHA‐specific B cells correlated with overall % H1 rHA‐specific B cells. These correlations infer the importance of IgG isotype and CD21^lo^CD27^+^ phenotype in reconstituting the rHA‐specific B‐cell level. Overall, HSCT recipients mounted robust HA‐antibody and B‐cell responses following IIV, comparable to HC, although we observed more interindividual heterogeneity among HSCT recipients.

### High HSCT responders to IIV had longer time intervals after transplantation

**Figure 4:**
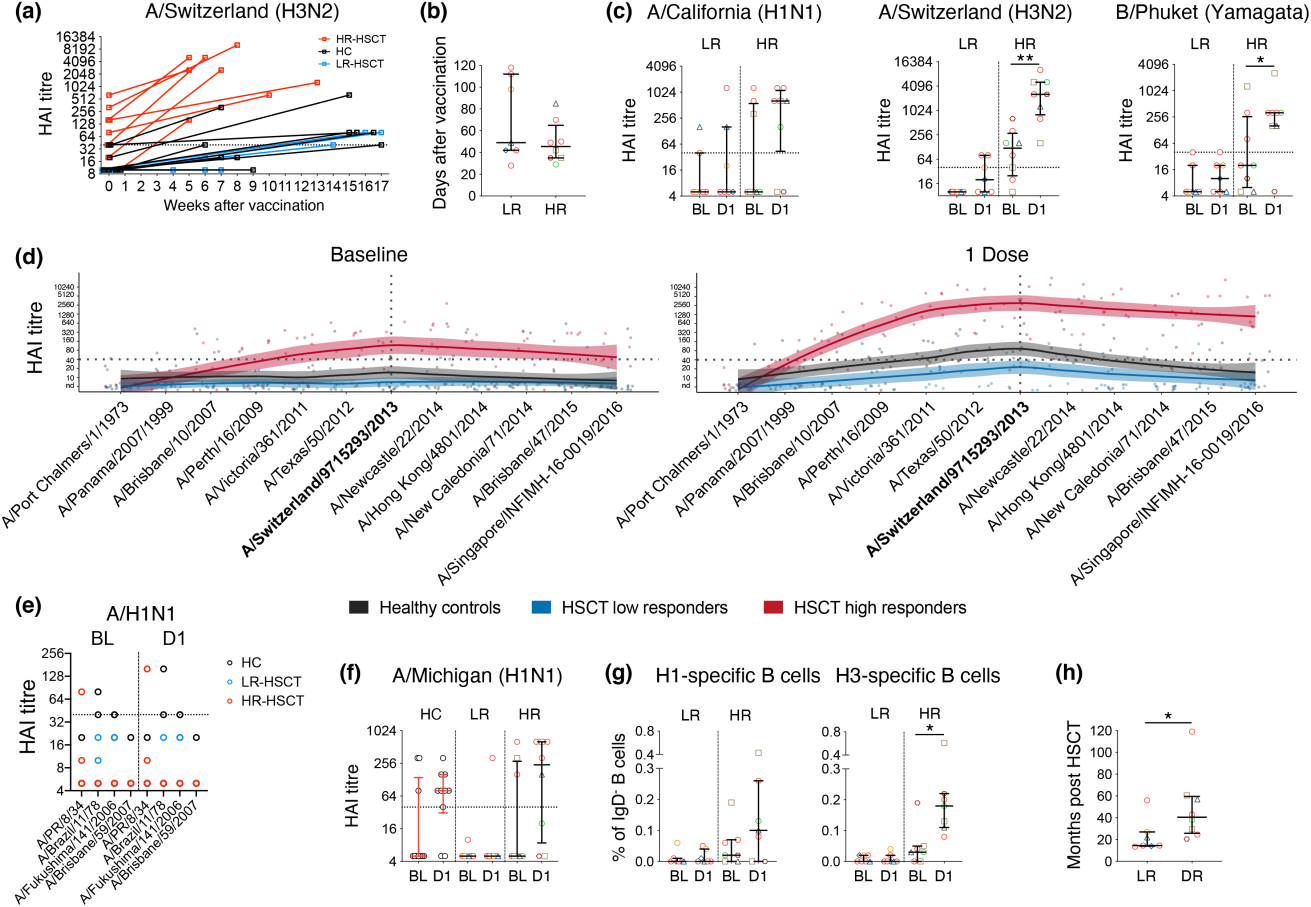
A subgroup of HSCT recipients had superior humoral responses towards A/H3N2. **(a)** HAI titre against A/Switzerland (H3N2) versus weeks after vaccination. **(b)** Days post vaccination per LR and HR group. **(c)** HAI titre against three vaccine strains. **(d)** HAI antibody landscapes against 12 A/H3N2 strains. Vertical and horizontal dotted lines indicate the vaccine strain and seroprotective HAI titre at 40 respectively. Solid lines indicate titre landscapes across strains estimated using GAMs and shadings indicate 95% CI for the model. Colour dots show individual participant titres against each antigen. **(e, f)** HAI titre against four pre‐pandemic A/H1N1 strains and A/Michigan (H1N1) strains. **(g)** Frequency of influenza‐specific B cells against H1 and H3 (n_HR‐HSCT_ = 7). **(h)** Time after transplantation for low and high responders of HSCT recipients. Bars indicate median and interquartile range. Symbols of HSCT recipients were the same as in Figure 1b. Technical replicates were not performed due to limited patient samples. Significance between baseline and after one dose was determined using the Wilcoxon test (n_HC_ = 10, n_LR‐HSCT_ = 7, n_HR‐HSCT_ = 8; *P < 0.05, **P < 0.01).

To understand the heterogeneity in humoral responses in HSCT recipients, we subdivided these participants based on the magnitude of their HAI response. The most prominent difference within the HSCT group was observed for post‐vaccination HAI titres against A/Switzerland (H3N2), with 53% of HSCT recipients (*n* = 8/15) having strikingly high HAI titres (up to 10 240), substantially higher than healthy controls (≤ 640) (Figure 4a). Thus, HSCT recipients were separated into two sub‐groups, a low A/H3N2 response group (LR‐HSCT; *n* = 7) and a high A/H3N2 response group (HR‐HSCT; *n* = 8), based on the median A/H3N2 HAI cut‐off after one IIV dose in the HSCT group (LR < 160, HR ≥ 160). Interestingly, all LR‐HSCT recipients had undetectable A/H3N2 HAI titres at baseline (< 20) compared to the HR‐HSCT group which had one recipient < 20, one at 20 and 6/8 having seroprotective titres ≥ 40 (Figure 4a). The timing of vaccination was not different between LR and HR groups (Figure 4b), which might have skewed the data if there was a waning of antibody responses with longer post‐vaccination sampling time intervals for LR patients; however, this was not the case. The HR‐HSCT group also had a higher median HAI titre post‐vaccination against A/H1N1 (median 5 (LR‐HSCT) and 640 (HR‐HSCT)) and B/Yamagata (median 10 (LR‐HSCT) and 320 (HR‐HSCT)) strains (Figure 4c).

To further characterise these high antibody responses from the HR‐HSCT group, HAI titres against 12 A/H3N2 strains were tested to construct antigenic landscapes (Figure 4d), including the vaccine strain (A/Switzerland/9715293/2013), two historical strains (A/Port Chalmers/1/1973 and A/Panama/2007/1999), eight recent vaccine strains (between 2007 and 2015), and one future unencountered strain from 2016 (A/Singapore/INFMH‐16‐0019/2016). At baseline, the HR‐HSCT group had a geometric mean titre (GMT) ≥ 40 HAI units against 8/12 strains, including the unencountered A/Singapore/2016 strain. Following vaccination, HAI titres against all strains, except the two historical strains, increased in the HR‐HSCT group with the largest magnitude among the three groups. GMT HAI titres of the HC group also increased following IIV but mainly targeting strains between 2011 and 2014. Conversely, GMTs of the LR‐HSCT group did not reach a seroprotective titre ≥ 40 for any H3N2 strains.

To determine whether high H3N2 responses were also associated with high titres towards H1N1 strains, HAI titres against five A/H1N1 strains, including four pre‐2009 pandemic strains (A/PR/8/34, A/Brazil/11/78, A/Fukushima/141/2006 and A/Brisbane/59/2007) and one post‐pandemic strain A/Michigan/45/2015 were tested. In fact, the majority of participants from all groups had low baseline HAI titres against the four pre‐2009 pandemic strains (< 40), and none were boosted at least fourfold following IIV (Figure 4e). HAI titre against post‐pandemic A/Michigan/45/2015 strain increased following IIV in both HC and HR‐HSCT groups, although not significantly but remained low in the LR‐HSCT group (Figure 4f).

Consistent with serological data, the frequency of H3‐specific B cells increased following IIV in the HR‐HSCT group (median 0.04% (BL) to 0.18% (D1), *P* = 0.0156), but not in the LR‐HSCT (Figure 4g) or HC group (Figure 3e). A similar trend was observed for H1‐specific B cells, albeit not statistically significant (Figure 4g). To further understand the differences between the HR‐HSCT and LR‐HSCT groups, we compared their available clinical data and demographics. Interestingly, individuals in the HR‐HSCT group had a longer time interval (median 40.5 months, *P* = 0.0193) post‐transplantation than the LR‐HSCT group (median 14.5 months; Figure 4h and Supplementary table 3).

Overall, IIV induced a broad antibody response with high magnitude towards A/H3N2 strains and the A/H1N1 pandemic strain in the HR‐HSCT group, while HC and LR‐HSCT antibody responses were mainly focused on the vaccine strain with lower magnitude. High responders to H3N2 were associated with increases in influenza‐specific B cells and longer time interval post‐transplantation.

### Systems serology approach reveals subclass of IgG1, IgG3 and Fc effector responses following IIV

**Figure 5:**
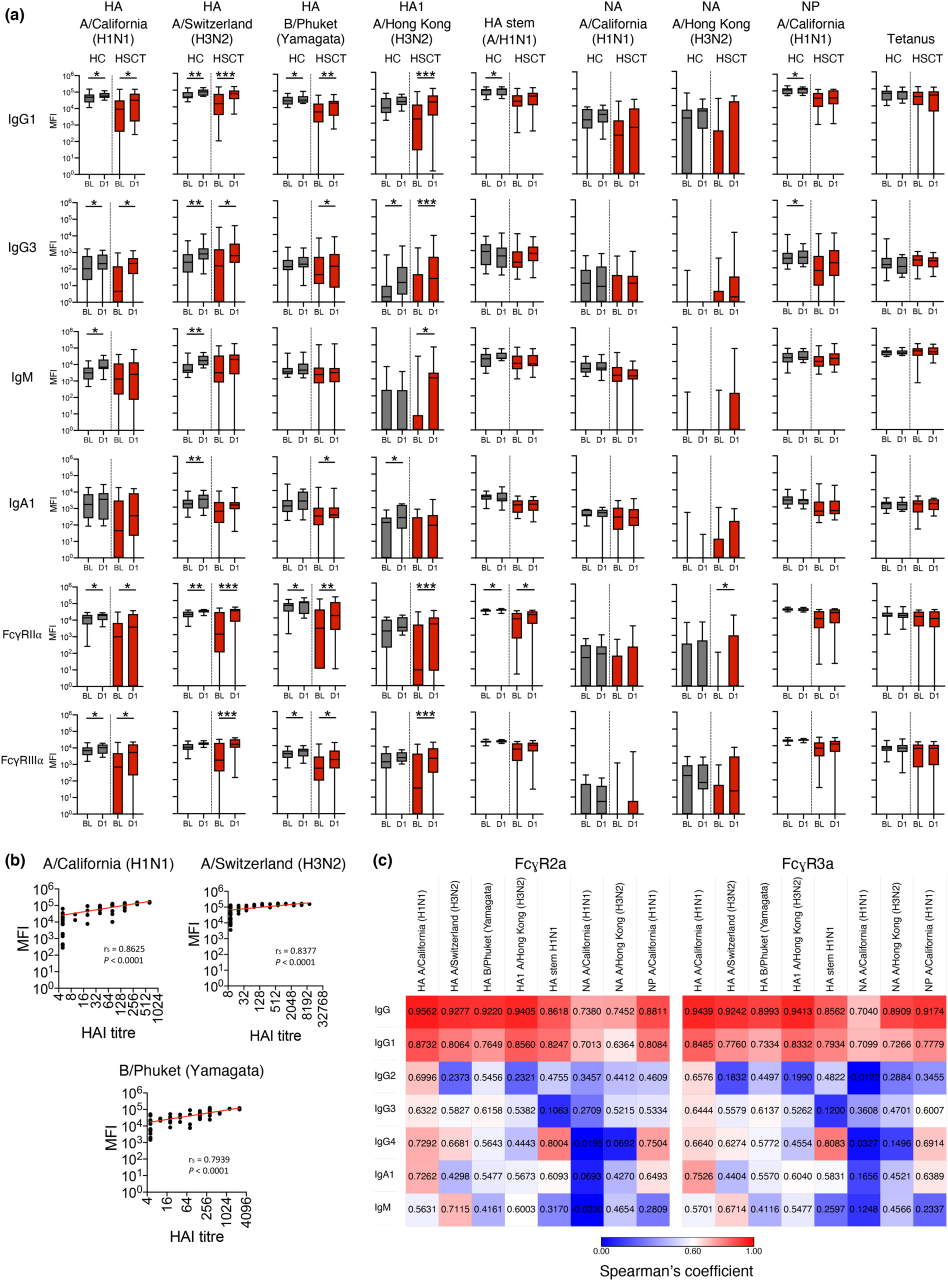
In‐depth analysis of antibody responses using multiplex bead assay. **(a)** Level of influenza‐specific antibodies for IgG1, IgG3, IgM, IgA1 and antibodies with Fc gamma receptor binding abilities. Boxes indicate median and interquartile range and whiskers indicate range. Significance between baseline and after one dose was determined using the Wilcoxon test for each group (n_HC_ = 10, n_HSCT_ = 15). *P < 0.05, **P < 0.01, ***P < 0.001. **(b)** Correlation between levels of HA‐specific IgG antibody (MFI value) and corresponding HAI titre. Correlation was assessed using Spearman's rank‐order correlation (n_HC_ = 14, n_HSCT_ = 18). **(c)** Spearman's correlation between antibody isotypes or IgG subclasses and Fc gamma receptor binding abilities for each influenza antigen (datapoints of HC and HSCT groups prior and post vaccination were combined for analyses). Technical replicates were not performed due to limited patient samples.

To gain further insights into the immune response to IIV in HSCT and to further compare the HR‐HSCT and LR‐HSCT groups, we performed in‐depth serological analyses using a multiplex antigen array^31^, ^32^ that included the three vaccine HA antigens (H1pdm09 A/California, H3 A/Switzerland, B/Phuket), an antigenically drifted H3 HA1 protein (A/HK/2014), an H1 HA stem antigen, two NA antigens (N1pdm09 A/California, N3 A/HK/2014) and NP (H1pdm09 A/California) (Figure 5). Tetanus toxoid and anti‐IgG antibody were included as controls. Antibodies for each antigen were assessed for IgG subclasses (IgG1, IgG2, IgG3, IgG4), IgM, IgA1 and their potential to engage Fc gamma receptors FcγRIIa (CD32a) and FcγRIIIa (CD16). Samples were assessed at a non‐saturating concentration (1:400) (Supplementary figure 4a and b) and no competition was observed from multiplexing (Supplementary figure 4c). Further validation showed strong (*r*_*S*_ > 0.86) and significant (*P* < 0.0001) correlations for IgG antibodies against all antigens between two independent experiments, except for negative anti‐IgG control where all the samples clustered highly together (Supplementary figure 5a).

Following IIV, IgG antibodies against influenza HA antigens increased in both HC and HSCT groups, which was specific for subclasses IgG1 and IgG3 but not IgG2 or IgG4 (Figure 5a and Supplementary figure 5b). Consistent with the H3N2 antibody landscape (Figure 4c), we observed broad IgG/IgG1 antibody responses post‐vaccination against the antigenically drifted A/Hong Kong/2014 strain in HSCT recipients but not the HC group. The HC group showed increased post‐vaccination responses in IgM and IgA1 targeting vaccine HA antigens and modest increases for IgG1 and IgG3 antibody responses against NP, but these were not observed in the HSCT group. Strong (*r*_*S*_ > 0.79) and significant (*P* < 0.0001) correlations were observed between IgG antibodies against the relevant HA and paired HAI titre for each vaccine strain (Figure 5b). Higher levels of vaccine HA‐specific antibodies engaging FcγRs were observed in both groups, which strongly correlated with levels of IgG and IgG1 (Figure 5c). Responses to NA antigens, however, were overall limited and the control tetanus‐specific antibodies and total IgG were not induced by IIV in either group.

Taken together, our multiplex data revealed that HSCT recipients mount strong IgG1 and IgG3 antibody responses to IIV vaccine antigens, with the potential to engage Fc receptors for effector function.

### High responders are immunologically distinct from low responders prior to IIV

**Figure 6:**
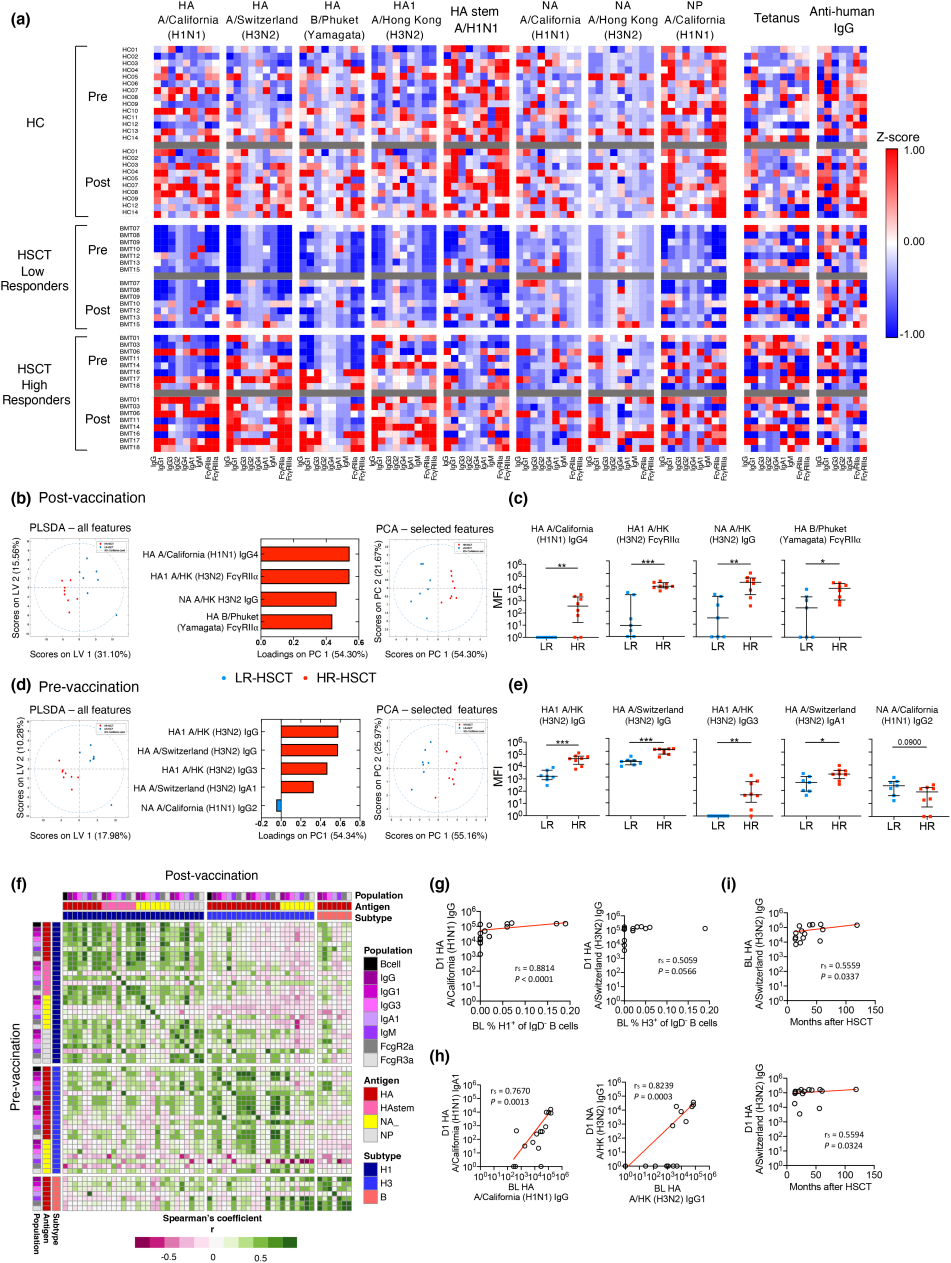
Multivariant analyses of HR‐HSCT and LR‐HSCT groups. **(a)** Levels of influenza‐specific antibodies separated for HC, LR‐HSCT and HR‐HSCT. Raw MFI data were standardised using *z*‐score before generating the heatmap. Value closer to 1 (i.e. hotter colour) indicates that the level of antibody was higher than other individuals, whereas value closer to −1 (blue) indicates lower antibody level **(b–e)** partial least squares discriminant analysis (PLS‐DA), loadings plot and principal component analysis (PCA) on antibody levels at dose 1 **(b)** or baseline **(d)** for LR‐HSCT and HR‐HSCT groups (10‐fold cross‐validation). PCA was performed with four and five selected features, respectively, to validate their importance in separating groups. **(c**, **e)** Direct comparisons of features are shown in loadings plots. Bars indicate median and interquartile range. Significance between the two groups was determined using the Mann–Whitney test (*P < 0.05, **P < 0.01, ***P < 0.001, ****P < 0.0001). **(f–i)** Correlations matrix **(f)** between pre‐vaccination and post‐vaccination influenza‐specific antibodies and B cells and **(g, h)** correlations of key features. **(i)** Correlations between A/Switzerland (H3N2) HA IgG and months after HSCT. Correlation was determined with Spearman's correlation. n_HC_ = 14, n_HSCT‐LR_ = 7, n_HSCT‐HR_ = 8. Technical replicates were not performed due to limited patient samples.

We subsequently visualised the multiplex antibody responses across antigens and isotypes between HC, HR‐ and LR‐HSCT groups via heatmap analysis. Consistent with HAI antibody and HA‐specific B‐cell response patterns, antibodies against the vaccine HA antigens generally increased post‐vaccination in the HR‐HSCT group but not the LR‐HSCT group, and were generally IgG1, IgG3, FcγRIIa and FcγRIIIa responses (Figure 6a).

To define distinct immunological features between HR‐ and LR‐HSCT groups post‐vaccination, we employed multivariate analyses combining baseline cellular data, HAI antibody and multiplex antigen array data, totalling 105 features from baseline (BL) and 72 features following IIV (D1). Strikingly, immune profiles of HR‐ and LR‐HSCT groups were clearly distinct following IIV, with enrichment of antibodies against HA IIV components in the HR‐HSCT group (Figure 6b). This set of four minimal distinct features was sufficient to separate high‐responder and low‐responder HSCT subgroups by an unsupervised principal component analysis (PCA) analysis, which was confirmed in univariate analyses (Figure 6c). Importantly, these selected features were not limited to the H3N2 component but included features representing all three vaccine components, indicating overall greater immune responses to IIV in high responders (HR‐HSCT).

Having shown that HR‐HSCT recipients have distinct immunological profiles post‐vaccination, we applied the same approach to the immune profiling of samples obtained prior to vaccination, to identify baseline immunological features that could predict the response post‐vaccination. Immunological profiles of HR‐ and LR‐HSCT groups prior to vaccination were clearly distinct (Figure 6d), with enrichment of antibodies against HA of various strains and isotypes (Figure 6d). Similar to post‐IIV, this set of five minimal distinct features was sufficient to separate the HR‐ and LR‐HSCT subgroups by unsupervised PCA analysis (Figure 6d), which was confirmed by univariate analyses (Figure 6e). These data indicate that HSCT recipients with higher responses post‐vaccination had greater pre‐existing A/H3N2‐specific immunity at baseline.

As the above analysis indicated clear baseline differences between the two groups, and as feature selection depicted a minimal set of features to avoid overfitting, we performed a correlation matrix of HA‐specific B cells and antibody measurements from the multiplex antigen array between pre‐ and post‐vaccination samples. Positive correlations (*r*_*S*_ > 0.5) were found between pre‐ and post‐vaccination levels of influenza‐specific immunity (Figure 6f). Indeed, the frequency of influenza‐specific B cells pre‐vaccination correlated with cognate antibody titres post‐vaccination, although this was more prominent for the H1 component (*r*_*S*_ = 0.8814, *P* < 0.0001; Figure 6g). Additionally, antibody levels pre‐vaccination positively (*r*_*S*_ > 0.7, *P* < 0.0013) correlated with post‐vaccination antibody levels for both H1 and H3 components (Figure 6h). Positive correlations were also observed between post‐transplantation time interval (months between transplantation and vaccination) and influenza‐specific antibodies at baseline or following IIV (Figure 6i).

Overall, our data indicate that high responders have distinct influenza‐specific immune profiles at baseline, which is associated with longer time interval post‐transplantation and stronger influenza‐specific immune responses to IIV. Whether the high responses observed are also related to imprinting effects from the donor is a possibility.

### Two doses of IIV can boost the humoral responses minorly in low responders

**Figure 7:**
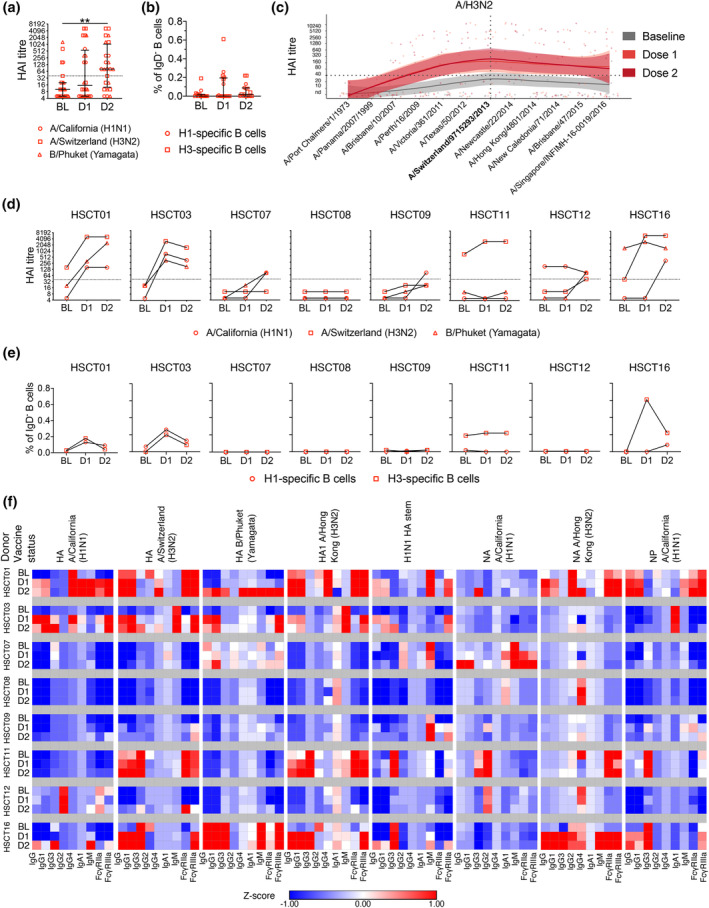
Humoral responses in HSCT recipients following two IIV doses. **(a, b)** Pooled analyses of HAI titre and influenza‐specific B‐cell frequency. Bars indicate the median and interquartile range. Significance was determined using the Friedman test followed by Dunn's multiple comparisons test (*n* = 8; **P < 0.01). **(c)** HAI antibody landscapes against 12 A/H3N2 strains of HSCT recipients that received two IIV doses. Vertical and horizontal dotted lines indicate the vaccine strain and the seroprotective HAI titre at 40 respectively. Solid lines indicate titre landscapes across strains estimated using GAMs and shadings indicate 95% CI for the model. Colour dots show individual participant titres against each antigen. **(d)** HAI titre and **(e)** influenza‐specific B‐cell frequency against the vaccine strains of each individual who received two IIV doses. **(f)** Heatmap of isotype distribution and Fc gamma receptor binding ability of influenza‐specific antibodies for each HSCT recipient that received two IIV doses. MFI values were standardised using *z*‐score. A value closer to 1 (i.e. a hotter colour) indicates that the level of antibody was higher than other individuals/time points. Note that participant HSCT01 had a documented influenza B virus infection between the administration of the second vaccine dose and the last blood drawing. Technical replicates were not performed due to limited patient samples.

Our data so far indicate that while some HSCT recipients mount robust immunity to IIV, others have no detectable response. To determine whether these non‐responders can be boosted by a second dose of IIV, we analysed humoral responses induced by two IIV doses (D2 time point, > 4 weeks after the second dose) in a smaller subset of HSCT recipients (*n* = 8). When analysing pooled data from three IIV components, HAI titres increased eight–fold after two IIV doses (median 10 (BL) to 80 (D2), *P* = 0.0035), which was not increased after the first dose compared to baseline (Figure 7a). Influenza‐specific B cells and HAI‐antibodies against other non‐vaccine A/H3N2 strains were not boosted by the second dose (Figure 7b and c). Interestingly, individuals who responded to the first dose did not mount enhanced HAI antibody responses to the second IIV dose. For individuals who did not respond to the first IIV dose, four of six did respond to the second dose for at least one of the antigens, albeit with modest increases (Figure 7d). Similarly, influenza‐specific B cells were not boosted after the second dose (Figure 7e). It is interesting to note that, in the multiplex array data, a fraction of low‐responding HSCT recipients did generate specific antibody responses to some influenza antigens following two IIV doses, or responses were moderately boosted from the first dose (Figure 7f). This was despite one participant (HSCT01) having documented influenza B virus infection between administration of the second vaccine dose and post‐second IIV blood sample, which lead to a large increase in B/Phuket HA‐specific antibodies between baseline and second IIV dose. Therefore, a single IIV dose is sufficient for HSCT recipients capable of mounting robust influenza‐specific immunity; however, a two‐dose regimen boosted the immunity in low responders minorly.

## Discussion

HSCT recipients, as a group of immunocompromised individuals, can have impaired immune responses towards IIV.^17^ However, the underlying mechanisms are far from being clear. A better understanding of immune responses towards IIV is essential for better vaccine strategies in HSCT recipients. We investigated humoral responses towards IIV and revealed that HSCT recipients had a different distribution of T‐ and B‐cell subsets compared with healthy controls prior to vaccination. HSCT recipients mounted a robust humoral response following IIV, including HAI antibodies, HA‐specific IgG1/IgG3/Fc receptor antibodies and influenza‐specific B‐cell responses, which were comparable to healthy controls. We highlight the importance of pre‐existing immunity in generating robust vaccine responses in HSCT recipients. Finally, a single IIV dose was sufficient for HSCT recipients capable of mounting robust influenza‐specific immunity; however, a two‐dose regimen was able to boost immune responses minorly, particularly in low responders.

Given the possible immunocompromised status in the HSCT recipients, we first profiled their immune reconstitution by assessing a broad range of innate and adaptive immune cells. In our HSCT group, a low CD4^+^ T‐cell frequency was found, while the frequency of CD8^+^ memory‐like T cells was higher, which is consistent with another HSCT study.^33^ Early after HSCT, T cells reconstitute through hyperproliferating of peripheral T cells.^34^ CD8^+^ T cells, especially memory populations, reconstitute earlier since they are less dependent on MHC molecules, while the CD4^+^ T‐cell compartment relies more on the regeneration of naïve T cells in the thymus, which is often deficient in adulthood.^5^, ^35^, ^36^, ^37^, ^38^, ^39^ In contrast, while B‐cell frequency was comparable between HC and HSCT groups, we observed a higher frequency of CD21^hi^CD27^−^ B cells and a lower frequency of CD21^hi^CD27^+^ and CD21^lo^CD27^+^ B cells in HSCT recipients, consistent with previous reports in both paediatric and adult HSCT recipients.^33^, ^40^ Our B‐cell findings can be explained by reconstitution of B cells being more dependent on *de novo* regeneration of naïve B cells in the bone marrow. It is also speculated that isotype‐switched or memory B cells are reduced due to limited antigen encounters after transplantation and/or lack of CD4^+^ T cell help.^26^ However, due to the oligoclonal expansion of memory T cells and *de novo* regeneration of naïve B cells, HSCT recipients may potentially have a narrower TCR repertoire and lack of B‐cell memory, which might contribute to the variable immune responses in our HSCT group.^5^, ^26^, ^33^, ^41^

Studies have investigated the immune responses of HSCT recipients towards different types of IIVs, such as ‘standard’, adjuvanted or high dose IIV. Due to differences in vaccine types and cohort characteristics, the reported immune responses vary considerably. Although it was reported that a HAI titre higher than 40 was needed in children to reach 50% protection,^42^ seroprotection and seroconversion rates (response at least fourfold higher than baseline) are still commonly used in most influenza vaccine studies of HSCT cohorts. Two studies have previously investigated immune responses towards trivalent influenza vaccine (TIV) in HSCT recipients.^25^, ^43^ While we found a similar seroprotection rate for A/H1N1 (53.3%) as in Fukatsu *et al*. study (59.3%),^43^ our HSCT group had a higher seroprotection rate for A/H3N2 and IBV (our study: 73.3% and 53.3% versus Fukatsu: 59.3% and 14.8% respectively). Interestingly, our HSCT group also had a higher seroconversion rate for A/H3N2 strain (66.7%), compared to 44.4%^43^ or 39%^25^ as reported. Similarly, IIV also induced influenza‐specific B‐cell responses in our HSCT group, as measured by previously established rHA probes.^27^, ^28^, ^29^ IgG^+^ B cells and CD21^hi^CD27^+^ B cells were the predominant isotype and phenotype in rHA^+^ B cells at baseline in both groups, while the frequency of total H1/H3 rHA‐specific B cells, IgG^+^rHA^+^ subset and CD21^lo^CD27^+^ rHA^+^ B cells all increased significantly after vaccination, which is consistent with our previous study in a larger healthy cohort.^29^

Regarding our multiplex antibody data, we found that the rise of HA‐specific IgG1 antibody was predominant in both HC and HSCT groups, consistent with previous studies using ELISA.^44^, ^45^, ^46^ Conversely, different levels of HA‐specific IgG2, IgG3 and IgG4 antibodies were observed in previous studies.^44^, ^46^ In our study, HA‐specific IgG3 antibodies were detectable in most participants and their levels increased significantly after vaccination, while IgG2 and IgG4 were not boosted by IIV. Apart from the neutralisation of pathogens, antibodies with the ability of binding Fc receptors are an important component of protective immunity towards influenza virus infection.^47^ Different antibody isotypes have variable potency of activating effector functions. For instance, IgG1 and IgG3 antibodies can bind to FcγRII and FcγRIII and are potent activators of Fc‐mediated effector functions such as antibody‐dependent cell‐mediated cytotoxicity and antibody‐dependent cell‐mediated phagocytosis.^47^, ^48^, ^49^ Similarly, we observed that influenza vaccine strain HA‐specific antibodies with FcγRIIa‐ or FcγRIIIa‐binding abilities both increased significantly, consistent with those found in healthy and HIV‐infected individuals following IIV.^50^ Several studies have suggested a stronger correlation between IgG3 and IgG1 subclasses with FcγR‐binding than the IgG2 and IgG4 subclasses,^51^, ^52^, ^53^ while IgG1 was the main isotype that correlated with FcγR binding for all antigens in our current study. The correlation of IgG1 with FcγR binding for non‐HA antigens also suggests FcγR‐binding ability by non‐neutralising antibodies, as previously reported.^47^ While correlates of protection from infections have mostly been investigated using HAI titres, it has also been reported that FcγR^−/−^ mice with similar antibody levels to wild‐type mice were more highly susceptible to influenza infection.^54^ Thus, effector functions of antibodies might be important for HSCT recipients, especially when direct neutralisation of the virus is suboptimal.

A subset of HSCT recipients (high responder, HR‐HSCT group) had a superior humoral immune response towards IIV compared to low responders (LR‐HSCT group) and healthy individuals (HC group), especially for the A/H3N2 strain. These high responders were vaccinated at longer time intervals after transplantation, therefore potentially more time for immune reconstitution which might explain why they responded more efficiently than the LR‐HSCT group. Studies also reported that longer time interval post‐transplantation positively correlated with a higher seroprotection rate^21^, ^24^, ^55^ or seroconversion rate.^16^, ^25^ Although these studies compared between HSCT recipients within or after their first‐year post‐transplantation, time after transplantation may still affect immune responses in HSCT recipients 1 year after transplantation, given the long time required for adaptive immunity to reconstitute.

Interestingly, HR‐HSCT recipients had high levels of HAI antibodies against numerous H3N2 strains from different clades, including vaccine clade 3C.3a (A/Switzerland/9715293/2013), 3C.2a (A/Hong Kong/4801/2014, A/New Caledonia/71/2014, A/Brisbane/47/2015, A/Singapore/INFIMH‐16‐0019/2016) and 3C.1 (A/Victoria/361/2011, A/Texas/50/2012). Similar broad antibody reactivity has been reported and studied by Kosikova *et al*.^56^ The authors found that a group of influenza vaccinees with > 40 pre‐vaccination HAI titre and > 16‐fold change in HAI titre post‐vaccination had antibodies with broader reactivity towards several drifted H3N2 strains. Using the ferret model, it was also demonstrated that sequential infections with H3N2 viruses from different clades improve avidity and cross‐reactivity of antibody responses towards all encountered strains.^56^

Our multivariant analyses revealed that HR‐HSCT recipients had higher baseline immunity towards A/Switzerland (H3N2) and A/California (H1N1), indicating HSCT recipients with pre‐existing immunity could have better vaccine responses. Although documented influenza virus infection or vaccination of HSCT recipients did not show significant differences, it is possible that high responders have more time to encounter a natural A/H3N2 influenza virus infection as they had a longer time interval after HSCT. On the other hand, memory B cells from donors may expand upon encountering corresponding antigens. Ambati *et al*.^57^ investigated the influenza vaccine response in HSCT recipients 6‐month post‐transplantation, whose donors received IIV prior to donation. Although vaccination of donors did not correlate with a better vaccine response in recipients, 6 months might be too short for full immune reconstitution, hence leading to suboptimal immune responses. Overall, high responders in HSCT recipients displayed greater humoral response towards IIV, likely as a result of pre‐existing immune memory.

Several studies have reported an increased seroprotection and/or seroconversion rate after two doses of the adjuvanted monovalent A/H1N1pdm09 vaccine.^22^, ^23^, ^24^ However, the improved humoral response was likely due to the low baseline antibody level (novel H1N1 strain). In contrast, many HSCT recipients in the current study already had detectable baseline antibody levels towards at least one vaccine strain, which peaked after the first IIV dose rather than the second dose. Similarly, Karras *et al*.^25^ found no differences in seroprotection or seroconversion rates after two doses of unadjuvanted trivalent IIV compared to one dose, even in HSCT recipients that were 1‐year post‐transplantation. We also found that antibody responses and FcγR‐binding ability of HSCT recipients were overall not boosted by the second IIV dose, although we observed modest incremental increases in antibody responses for a few low‐responder HSCT recipients.

The current study has some limitations. We acknowledge the relatively small number of participants and that blood samples were not obtained at all time points for some participants; however, comparisons between baseline and post‐IIV were performed using paired data. Given the smaller cohort size, we were then able to perform comprehensive analyses for all the participants and present data per patient, particularly for the second dose analyses. We also acknowledge that while the proportion of T and B cells within the PBMC population was different in HSCT recipients compared to healthy controls, HSCT recipients may have an impairment in immune cellular function, such as clinical complications needing immunosuppressive treatment early on within 6–12 months post‐transplantation, which can affect their subsequent immune responses.

In conclusion, our study showed that immune memory is important for robust vaccine response in HSCT recipients. HSCT recipients at longer time interval post‐transplantation displayed a more robust humoral response compared to low responders and perhaps two doses of IIV can improve immune responses in low responders. However, as most of the low responders did not respond to the second dose, vaccination regimens such as mRNA vaccines that boost CD8^+^ T‐cell responses might benefit HSCT recipients.

## Methods

### Study participants and ethics statement

Participants in this study were recruited from the Alfred Hospital between May and August 2015. Experiments conformed to the Declaration of Helsinki Principles and the Australian National Health and Medical Research Council Code of Practice. The study was approved by the Alfred Hospital (#226/14) and the University of Melbourne Ethics Committee (#1442644). Signed informed consent was obtained from all participants. Individuals who had already been vaccinated in the same season or had any contraindication to influenza vaccination (e.g. egg allergy) were excluded. Adult outpatients (18–65 years of age) who received allogenic HSCT more than 12 months previously and did not receive intravenous immunoglobulin within the previous 3 months were eligible for enrolment. In Australia, influenza vaccination is recommended for all HSCT recipients 6 months after HSCT, so patients enrolled in this study were scheduled to receive IIV during their usual healthcare follow‐up. The HC group included healthy adult volunteers (18–60 years of age) who were scheduled to have a single dose of IIV, did not have chronic renal failure or asplenia and were not immunosuppressed. The IIV used in this study was Fluvax® (BioCSL, Parkville, Australia), which contains 15 μg of HA from each of the three influenza strains: A/California/07/2009 (H1N1) pdm09–like virus, A/Switzerland/9715293/2013 (H3N2)–like virus and B/Phuket/3073/2013–like virus (Yamagata lineage). Blood samples in serum red cap tubes were centrifuged and sera were collected. The Ficoll‐Paque separation was performed to isolate PBMCs from heparinised whole blood and cryopreserved.

### Cytokine measurement by cytometric bead array (CBA)

Seventeen cytokines (IL‐1β, IL‐2, IL‐4, IL‐6, IL‐8, IL‐10, IL‐12p70, IL‐17α, TNF, CD178/FasL, IFN‐α, IFN‐γ, MCP‐1, MIP‐1α, MIP‐1β, RANTES and Granzyme B) were analysed using a human CBA Flex Sets (BD Biosciences, San Jose, USA) following manufacturer's instructions, but scaled down to using 1/10 of reagent and sample volumes. Serum samples were diluted 1:100 for the detection of RANTES or 1:4 for all the other cytokines in the assay diluent. Samples were acquired on a BD FACSCanto II cytometer (BD Bioscience, San Jose, USA) and analysed with the FCAP Array software (BD Bioscience, San Jose, USA).

### Flow cytometry

PBMCs were stained for 30 min on ice with corresponding antibody panels (Supplementary tables 4 and 5). After fixing in 1% PFA, stained cells were acquired on a LSR Fortessa II (BD Bioscience, San Jose, USA) and data were analysed using FlowJo 10 (FlowJo, LLC, Ashland, USA). Staining for influenza‐specific B cells was performed as previously described on cryopreserved PBMCs.^29^, ^30^

### HAI assay

Haemagglutination inhibition assays were performed at the WHO Collaborating Centre for Influenza (VIC, Australia) following WHO guidelines. Briefly, 25 μL of receptor‐destroying enzyme‐treated serum samples were twofold serially diluted, starting at 1:10 dilution for A/H1N1 and IBV or 1:20 dilution for A/H3N2. Twenty‐five microlitres of relevant influenza virus at 4 HA units was added to each well for 45–60 min at room temperature. Twenty‐five microlitres of 1% turkey (A/H1N1, IBV) or guinea pig (A/H3N2) RBCs was added for another 30 min or 45–60 min respectively. Results were analysed by eye and HAI titre defined as reciprocal of the highest dilution of serum that completely inhibits haemagglutination, as seen by teardrop or button of pelleted RBC. HAI titre of 5 or 10 was assigned to individuals with non‐detectable HAI titre for A/H1N1 and IBV strains (< 10) or A/H3N2 strain (< 20), respectively, for calculation. A HAI titre ≥ 40 was defined as seroprotective and seroconversion was defined as ≥ fourfold increase in HAI titre. Antibody landscapes were constructed using generalised additive models (GAMs) to fit log_10_ HAI titres. The GAM function from the R packages mgcv and ggplot2 was used to generate the plots and compare landscapes.

### Multiplex bead assay

The multiplex bead assay was adapted from Chung *et al*.^31^ Briefly, magnetic microspheres (Luminex, Austin, USA) from 12 different bead ‘regions’ were selected and each was coupled with influenza or control proteins. Before staining, the antigen‐coupled microspheres were mixed to achieve 500 beads per sample per bead ‘region’ and topped up with 1% BSA/PBS to achieve 50 μL of bead mixture per sample. Fifty microlitres of bead mixture and 50 μL of 1:200 diluted serum were added into a 96‐well flat‐bottom microplate and the plate was incubated at 4°C overnight. The plate was then washed three times by a plate washer (BioTek, Winooski, USA). For each plate, one of the seven PE‐conjugated detector antibodies (IgG, IgG1‐4, IgA1, IgM; all from Southern Biotech, Birmingham, USA) or the two FcγR dimers (FcγRIIa (H131) and FcγRIIIa (V158); generated and provided by Prof Mark Hogarth (Burnet Institute, VIC, Australia)) were diluted to a final concentration of 1.3 μg mL^−1^, and 50 μL of the respective antibody/dimer was added to each well. The plate was incubated for a further 2 h at RT. For the two FcγR dimers, 50 μL of 1.3 μg mL^−1^ streptavidin‐PE (Invitrogen, Carlsbad, USA) was added after washing and the plate was incubated for a further hour at RT. After the final wash, the beads were resuspended in 100 μL of sheath fluid and incubated for 10 min at RT. Finally, the plate was read by the FlexMap 3D machine and data were analysed using the xPonent software (both by Luminex, Austin, USA). All incubations in this assay were performed on a plate shaker. For comparisons across different proteins and detector antibodies, all data were log‐transformed using the following equation, where *x* is the right‐shifted data and *y* is the log‐transformed data: *y* = log_10_(*x* + 1). This process transformed the features into being normally distributed. In all subsequent multivariate analyses, the data were normalised by mean centring and variance scaling each feature using the *z* score. Principal component analysis (PCA) and partial least squares discriminant analysis (PLS‐DA) were performed using MATLAB (MathWorks, Natick, USA). Lasso regression was performed following PLS‐DA, frequency of selected features in resampling was considered as the criterion of variable importance. The resampling of the data was performed via fourfold, fivefold, sixfold, sevenfold, eightfold, ninefold, 10‐fold, 11‐fold, 12‐fold, 13‐fold, 14‐fold and 15‐fold cross‐validation, all showing the four/five features to be most important in separating groups. Following model prediction, 10‐fold cross‐validation (CV) was selected since it was the most consistent. It was repeated 1000 times by randomly assigning the observations, and the result is shown in the figure. Heatmaps for visualisation were generated using the online Morpheus heatmap software (https://software.broadinstitute.org/morpheus; the Broad Institute, MA, USA). Except for the heatmap in Figure 6f, which was generated in R using the Hmisc (https://CRAN.R-project.org/package=Hmisc) and pheatmap (https://CRAN.R-project.org/package=pheatmap) packages.

### Statistical analyses

All statistical tests were performed using Prism 8 and 9 (GraphPad, Boston, USA). For non‐parametric unpaired or paired datasets, the Mann–Whitney *U*‐test or the Wilcoxon test were used for comparisons respectively. Spearman's rank‐order correlation was used to analyse the correlation for non‐parametric data. Correlation matrixes were generated in R using the Hmisc and corrplot packages. Non‐numerical data were grouped and analysed using Fisher's exact test. The significance levels were defined as *P < 0.05, **P < 0.01, ***P < 0.001 and ****P < 0.0001.

## AUTHOR CONTRIBUTIONS

**Wuji Zhang:** Data curation; formal analysis; investigation; writing – original draft; writing – review and editing. **Louise C Rowntree:** Data curation; formal analysis; investigation; writing – review and editing. **Ramona Muttucumaru:** Conceptualization; writing – review and editing. **Timon Damelang:** Formal analysis; investigation; methodology; writing – review and editing. **Malet Aban:** Formal analysis; investigation; writing – review and editing. **Aeron C Hurt:** Formal analysis; methodology; resources; writing – review and editing. **Maria Auladell:** Formal analysis; writing – review and editing. **Robyn Esterbauer:** Formal analysis; investigation; methodology. **Bruce Wines:** Methodology; resources. **Mark Hogarth:** Methodology; resources. **Stephen J Turner:** Conceptualization; investigation; writing – review and editing. **Adam K Wheatley:** Formal analysis; investigation; methodology; resources. **Stephen J Kent:** Methodology; resources; writing – review and editing. **Sushrut Patil:** Investigation; methodology. **Sharon Avery:** Methodology; project administration. **Orla Morrissey:** Methodology. **Amy W Chung:** Formal analysis; methodology; resources; supervision. **Marios Koutsakos:** Investigation; supervision; writing – review and editing. **Thi HO Nguyen:** Conceptualization; formal analysis; investigation; supervision; writing – original draft; writing – review and editing. **Allen C Cheng:** Conceptualization; investigation; methodology; project administration; resources; supervision; writing – review and editing. **Tom C Kotsimbos:** Conceptualization; investigation; methodology; project administration; resources; writing – review and editing. **Katherine Kedzierska:** Conceptualization; funding acquisition; methodology; resources; software; supervision; writing – original draft; writing – review and editing.

## Conflict of interest

MK has acted as a consultant for Sanofi group of companies but not at the time the research was conducted. All other authors have declared that no conflict of interest exists.

## Supporting information


Supplementary table 1
Supplementary table 2
Supplementary table 3
Supplementary table 4
Supplementary table 5
Supplementary figure 1
Supplementary figure 2
Supplementary figure 3
Supplementary figure 4
Supplementary figure 5

## Data Availability

All data generated or analysed during this study are included in this published article (and its Supplementary information files). All relevant data are also available from the authors upon request.
