# Supplementary material for: Robust immunity to influenza vaccination in haematopoietic stem cell transplant recipients following reconstitution of humoral and adaptive immunity
Source: Clin Transl Immunology. 2023 Jun 27;12(6):e1456. doi: 10.1002/cti2.1456 (PMC10294294; doi:10.1002/cti2.1456)
Supplement: Supplementary file 1 — Supplementary table 1 Supplementary table 2 Supplementary table 3 Supplementary table 4 Supplementary table 5 Supplementary figure 1 Supplementary figure 2 Supplementary figure 3 Supplementary figure 4 Supplementary figure 5 [file CTI2-12-e1456-s001.pdf]

Robust immunity to influenza vaccination in haematopoietic stem cell transplant recipients following reconstitution of humoral and adaptive immunity

Wuji Zhang[1], Louise C Rowntree[1], Ramona Muttucumaru[2], Timon Damelang[1], Malet Aban[3], Aeron C Hurt[3,4], Maria Auladell[1,5], Robyn Esterbauer[1], Bruce Wines[6], Mark Hogarth[6], Stephen J Turner[7], Adam K Wheatley[1], Stephen J Kent[1,8], Sushrut Patil[9], Sharon Avery[9], Orla Morrissey[2], Amy W Chung[1], Marios Koutsakos[1]*, Thi HO Nguyen[1]*, Allen C Cheng[10,11]*, Tom C Kotsimbos[12,13]* and Katherine Kedzierska[1,14]*

Supplementary Data

List of Supplementary tables

Supplementary table 1. Age and gender of the HC and HSCT participants
Supplementary table 2. Demographics of HSCT recipients at enrolment
Supplementary table 3. Demographics of Low and High A/H3N2 responders
Supplementary table 4. Flow cytometry antibody panel for PBMC subsets
Supplementary table 5. Antibody panel for B cell subsets and influenza-specific B cells

List of Supplementary figures

Supplementary figure 1. Cytokine and chemokine levels at enrollment.
Supplementary figure 2. Gating strategy for peripheral blood mononuclear cell subsets.
Supplementary figure 3. Gating strategy for B cell subsets and influenza-specific B cells.
Supplementary figure 4. Optimization of multiplex bead assay conditions.
Supplementary figure 5. Reproducibility of the multiplex bead assay.

Supplementary table 1. Age and gender of the HC and HSCT participants

	**HC** (n = 14)	**HSCT** (n = 18)	***P*-value**
Age at enrollment (years), median (range)	33 (18-57)	35 (21-65)	0.6125#
Female, n (%)	10 (71.4%)	7 (38.9%)	0.1493^

#Significance was determined using the Mann-Whitney test; ^Significance was determined using the Fisher's exact test. Age was unknown for two participants, one in each group. Gender was unknown for one HSCT participant.

Supplementary table 2. Demographics of HSCT recipients at enrolment

HSCT recipients		Total (n = 18)	One dose (n = 7)	Two doses (n = 8)	*P*-value
Age at enrolment (years), median (range)[Ω]		35 (21-65)	33 (21-52)	36.5 (23-50)	0.6751[#]
Age at transplantation (years), median (range)[Ω]		33 (17-61)	28 (17-50)	35 (22-47)	0.5907[#]
Female, n (%)[Ω]		7 (38.9%)	3 (42.9%)	4 (50%)	> 0.9999^
Ethnicity (%)	Australian	12 (70.6%)	4 (57.1%)	7 (87.5%)	0.2821[^, *]
	Indian	2 (11.1%)	1 (14.3%)	1 (12.5%)	
	English	1 (5.6%)	1 (14.3%)	0	
	Polish	1 (5.6%)	1 (14.3%)	0	
	Macedonian	1 (5.6%)	0	0	
	Unknown	1 (5.6%)	0	0	
Transplantation-to-vaccination interval (months), median (range)[Ω]		29.5 (13.5-119)	29.5 (14.5-119)	23.25 (13.5-60.5)	0.4443[#]
Past season influenza vaccination, n (%)	Yes	5 (27.8%)	3 (42.9%)	2 (25%)	0.6084[^, *]
	None documented	8 (44.4%)	4 (57.1%)	2 (25%)	
	Unknown	5 (27.8%)	0	4 (50%)	
Documented influenza infection, n (%)	Yes	2 (11.1%)	1 (14.3%)	1 (12.5%)	> 0.9999[^, *]
	None documented	14 (77.8%)	6 (85.7%)	6 (75%)	
	Unknown	2 (11.1%)	0	1 (12.5%)	
Underlying diseases, n (%)	ALL	2 (11.1%)	2 (28.6%)	0	0.6084[^, Φ]
	B-ALL	3 (16.7%)	1 (14.3%)	2 (25%)	
	T-ALL	2 (11.1%)	1 (14.3%)	1 (12.5%)	
	Acute myeloid leukemia	1 (5.6%)	0	1 (12.5%)	
	Blastic plasmacytoid DC neoplasm	1 (5.6%)	0	0	
	Chronic myeloid leukemia	1 (5.6%)	1 (14.3%)	0	
	Multiple myeloma	2 (11.1%)	1 (14.3%)	1 (12.5%)	
	Hodgkin's lymphoma	2 (11.1%)	1 (14.3%)	1 (12.5%)	
	Marginal zone lymphoma	1 (5.6%)	0	0	
	Myelodysplasia	1 (5.6%)	0	1 (12.5%)	
	Myelofibrosis	1 (5.6%)	0	1 (12.5%)	
	Unknown	1 (5.6%)	0	0	
Donor type, n (%)	HLA-identical sibling	9 (50%)	4 (57.1%)	4 (50%)	> 0.9999^
	Matched unrelated donor	8 (44.4%)	3 (42.9%)	4 (50%)	
	Unknown	1 (5.6%)	0	0	
Donor age (years), median (range)[†]		31 (15-53)	35.5 (15-53)	32 (26-49)	0.9452[#]
Conditioning regimen, n (%)	Myeloablative	7 (38.9%)	2 (28.6%)	4 (50%)	0.6084[^, *]
	Reduced intensity	3 (16.7%)	2 (28.6%)	1 (12.5%)	
	Nonmyeloablative	1 (5.6%)	0	1 (12.5%)	
	Unknown	7 (38.9%)	3 (42.9%)	2 (25%)	
Previous GvHD, n (%)	Yes	7 (38.9%)	2 (28.6%)	4 (50%)	0.6084[^, *]
	None documented	8 (44.4%)	4 (57.1%)	3 (37.5%)	
	Unknown	3 (16.7%)	1 (14.3%)	1 (12.5%)	
Ongoing GvHD, n (%)	Yes	4 (22.2%)	1 (14.3%)	3 (37.5%)	0.5692[^, *]
	None documented	11 (61.1%)	6 (85.7%)	3 (37.5%)	
	Unknown	3 (16.7%)	0	2 (25%)	
Immunosuppressive treatment, n (%)	None	5 (27.8%)	3 (42.9%)	2 (25%)	0.6084[^, *]
	Cyclosporin	3 (16.7%)	0	2 (25%)	
	Prednisolone	1 (5.6%)	0	1 (12.5%)	
	Cyclosporin + Prednisolone	1 (5.6%)	0	1 (12.5%)	
	Prednisolone + Tacrolimus	1 (5.6%)	0	1 (12.5%)	
	Brentuximab	1 (5.6%)	1 (14.3%)	0	
	Thalidomide	1 (5.6%)	1 (14.3%)	0	
	Bortezomib	1 (5.6%)	0	0	
	Unknown	4 (22.2%)	2 (28.6%)	1 (12.5%)	

[#]Significance was determined using the Mann-Whitney test; ^Significance was determined using the Fisher's exact test; *Comparison between the first row and combining the other rows; [Φ]Grouped by all leukemia and others; [Ω]Data were unknown for one participant; [†]Data were unknown for three participants. Significance was determined between HSCT recipients that received one or two IIV dose(s). ALL, acute lymphoblastic leukemia; GvHD, graft versus host disease.

Supplementary table 3. Demographics of Low and High A/H3N2 responders

Clinical data		Low responders (n = 7)	High responders (n = 8)	***P*-value**
Age at enrollment (years), median (range)		33 (21-52)	49 (21-50)	0.2171$^{\#}$
Age at transplantation (years), median (range)		29 (20-50)	42 (17-48)	0.2948$^{\#}$
Female, n (%)		3 (42.9%)	4 (50%)	> 0.9999^
Ethnicity (%)	Australian	5 (71.4%)	6 (75%)	> 0.9999^, *
	Indian	1 (14.2%)	1 (12.5%)	
	English	0	1 (12.5%)	
	Polish	1 (14.2%)	0	
Transplantation-to-vaccination interval (months), median (range)		14.5 (13.5-56)	40.5 (20.5-119)	0.0193$^{\#}$
Vaccination-to-blood collection interval (days), median (range)		49 (28-118)	45.5 (29-85)	0.4120$^{\#}$
Past season influenza vaccination, n (%)	Yes	2 (28.6%)	3 (37.5%)	> 0.9999^, *
	None documented	2 (28.6%)	4 (50%)	
	Unknown	3 (42.9%)	1 (12.5%)	
Documented influenza infection, n (%)	Yes	0	2 (25%)	0.4667^, *
	None documented	6 (85.7%)	6 (75%)	
	Unknown	1 (14.2%)	0	
Underlying diseases, n (%)	ALL	1 (14.2%)	1 (12.5%)	0.6084^, $^{\Phi}$
	B-ALL	3 (42.9%)	0	
	T-ALL	0	2 (25%)	
	Acute myeloid leukemia	0	1 (12.5%)	
	Chronic myeloid leukemia	1 (14.2%)	0	
	Multiple Myeloma	0	2 (25%)	
	Hodgkins Lymphoma	1 (14.2%)	1 (12.5%)	
	Myelodysplasia	1 (14.2%)	0	
	Myelofibrosis	0	1 (12.5%)	
Donor type, n (%)	HLA-identical sibiling	3 (42.9%)	5 (62.5%)	0.6193^
	Matched unrelated donor	4 (57.1%)	3 (37.5%)	
Donor age (years), median (range)		28.5 (20-53)	45 (15-52)	0.3660$^{\#}$
Conditioning regimen, n (%)	Myeloablative	4 (57.1%)	2 (25%)	0.3147^, *
	Reduced intensity	0	3 (37.5%)	
	Nonmyeloablative	1 (14.2%)	0	
	Unknown	2 (28.6%)	3 (37.5%)	
Previous GvHD, n (%)	Yes	3 (42.9%)	3 (37.5%)	> 0.9999^, *
	None documented	3 (42.9%)	4 (50%)	
	Unknown	1 (14.2%)	1 (12.5%)	
Ongoing GvHD, n (%)	Yes	1 (14.2%)	2 (25%)	> 0.9999^, *
	None documented	4 (57.1%)	6 (75%)	
	Unknown	2 (28.6%)	0	
Immunosuppresive treatment, n (%)	None	4 (57.1%)	1 (12.5%)	0.3147^, *
	Cyclosporin	1 (14.2%)	1 (12.5%)	
	Prednisolone	0	1 (12.5%)	
	Cyclosporin + Prednisolone	0	1 (12.5%)	
	Prednisolone + Tacrolimus	1 (14.2%)	0	
	Brentuximab	0	1 (12.5%)	
	Thalidomide	0	1 (12.5%)	
	Unknown	1 (14.2%)	2 (25%)	

$^{\#}$Significance was determined using the Mann-Whitney test; ^Significance was determined using the Fisher's exact test; *Comparison between the first row and combining the other rows; $^{\Phi}$Grouped by all leukemia and others. ALL, acute lymphoblastic leukemia; GvHD, graft versus host disease.

Supplementary table 4. Flow cytometry antibody panel for PBMC subsets

Antibody	Clone	Fluorochrome	Dilution	Vendor
γδ-TCR	11F2	FITC	1:30	BD
CD8	SK1	PerCP-Cy5.5	1:200	BD Pharmingen
CD3	UCHT1	APC	1:100	eBioscience, CA, USA
CD16	3G8	Alexa Fluor 700	1:100	BioLegend, CA, USA
CD45RA	HI100	APC-H7	1:50	BD Pharmingen
HLA-DR	L243	V450	1:100	BD
Live/Dead		Aqua	1:500	Molecular Probes, Life Technologies, CA, USA
CD161	HP-3G10	BV605	1:50	BioLegend
CD4	SK3	BV650	1:200	BD Horizon
CD27	L128	BV711	1:100	BD Horizon
CD56	NCAM16.2	BV786	1:100	BD Horizon
TCRVα7.2	3C1D	PE	1:400	BioLegend
CD19	J3-199	ECD	1:100	Beckman Coulter, CA, USA
CD14	MΦP9	PE-Cy7	1:50	BD Pharmingen

Supplementary table 5. Antibody panel for B cell subsets and influenza-specific B cells

Antibody	Clone	Fluorochrome	Dilution	Vendor
H3 rHA	N/A	APC	N/A	Made in-house
CD20	2H7	Alexa Fluor 700	1:150	BD
IgM	G20-127	BUV395	1:150	BD
CD21	B-ly4	BUV737	1:300	BD
BPHU		BV421	N/A	In-house
Free SA		BV510	1:600	BD Horizon
Live/Dead		Aqua	1:500	Molecular Probes
CD3	OKT3	BV510	1:600	BioLegend
CD8	RPA-T8	BV510	1:1500	BioLegend
CD10	HI10a	BV510	1:750	BioLegend
CD14	M5E2	BV510	1:300	BioLegend
CD16	3G8	BV510	1:500	BioLegend
CD27	O323	BV605	1:150	BioLegend
IgG	G18-145	BV786	1:75	BD
H1 rHA	N/A	PE	N/A	Made in-house
CD19	J3-119	ECD	1:150	Beckman
IgD	IA6-2	PE-Cy7	1:500	BD

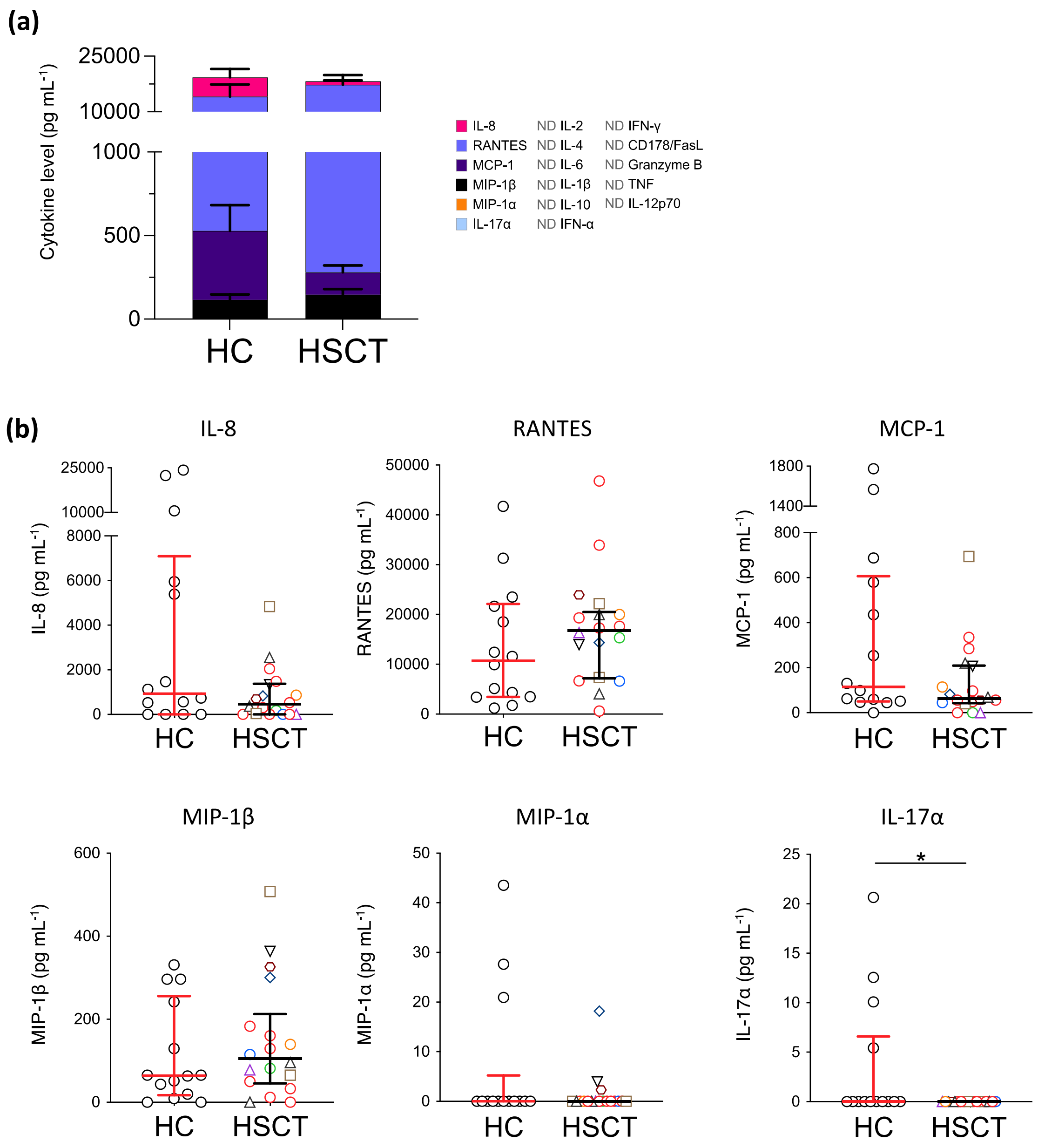

Supplementary figure 1. Cytokine and chemokine levels at enrollment. The levels of 17 cytokines in serum collected at enrollment were measured for both healthy control (n_{HC} = 14) and HSCT (n_{HSCT} = 18) groups using cytometric bead array. **(a)** Stacked bar graph indicates mean concentration of cytokines (+ SEM). Cytokines and chemokines shown as ND had a non-detectable concentration below 10 pg ml^{-1}. **(b)** Serum concentration of IL-8, RANTES, MCP-1, MIP-1β, MIP-1α, and IL-17α. Bars indicate the median and interquartile range. Symbols of HSCT recipients were the same as in Figure **1b**. Technical replicates were not performed due to limited patient samples. Statistical significance between the two groups was determined using the Mann-Whitney *U*-test (**P* < 0.05).

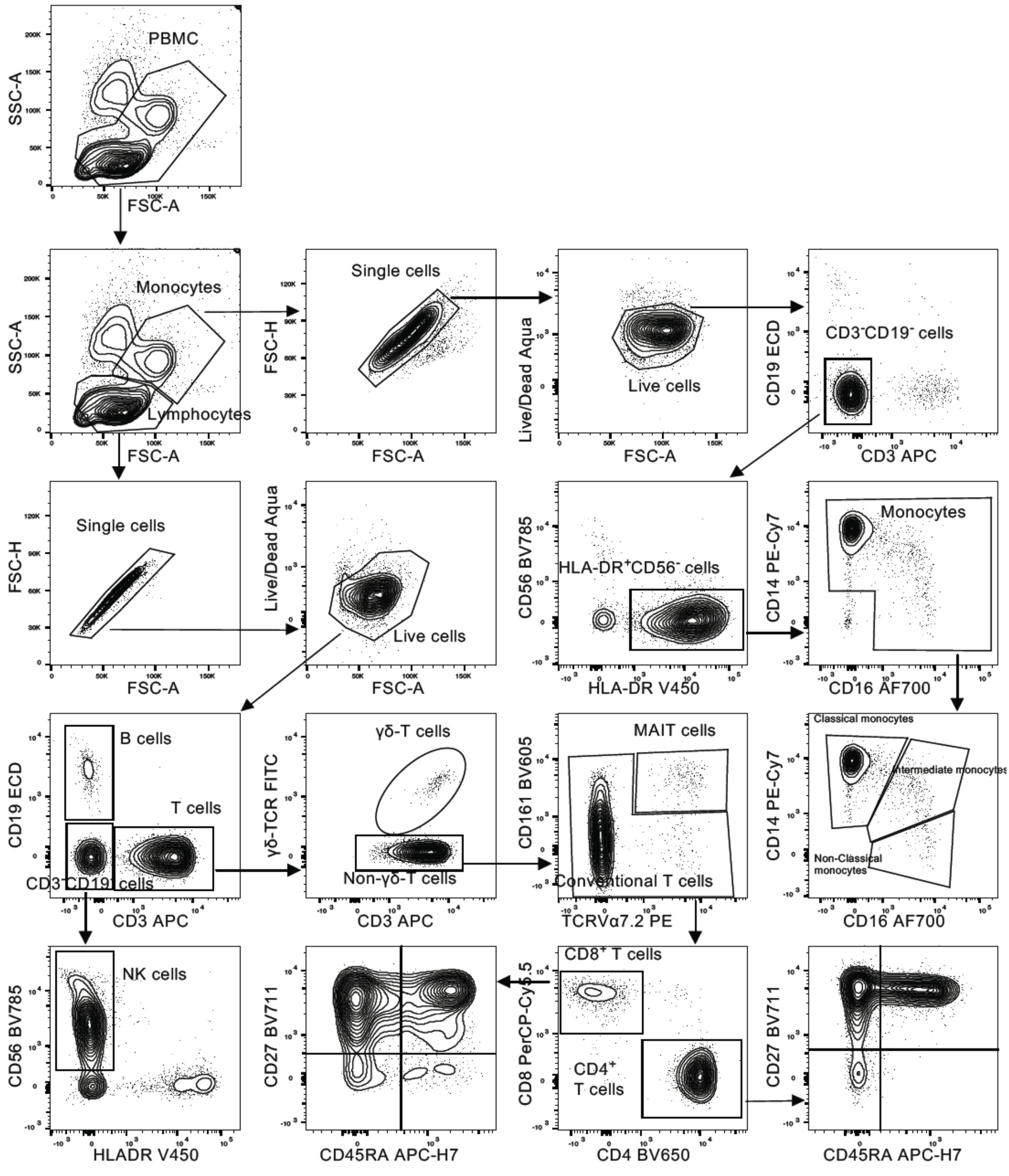

Supplementary figure 2. Gating strategy for peripheral blood mononuclear cell subsets.

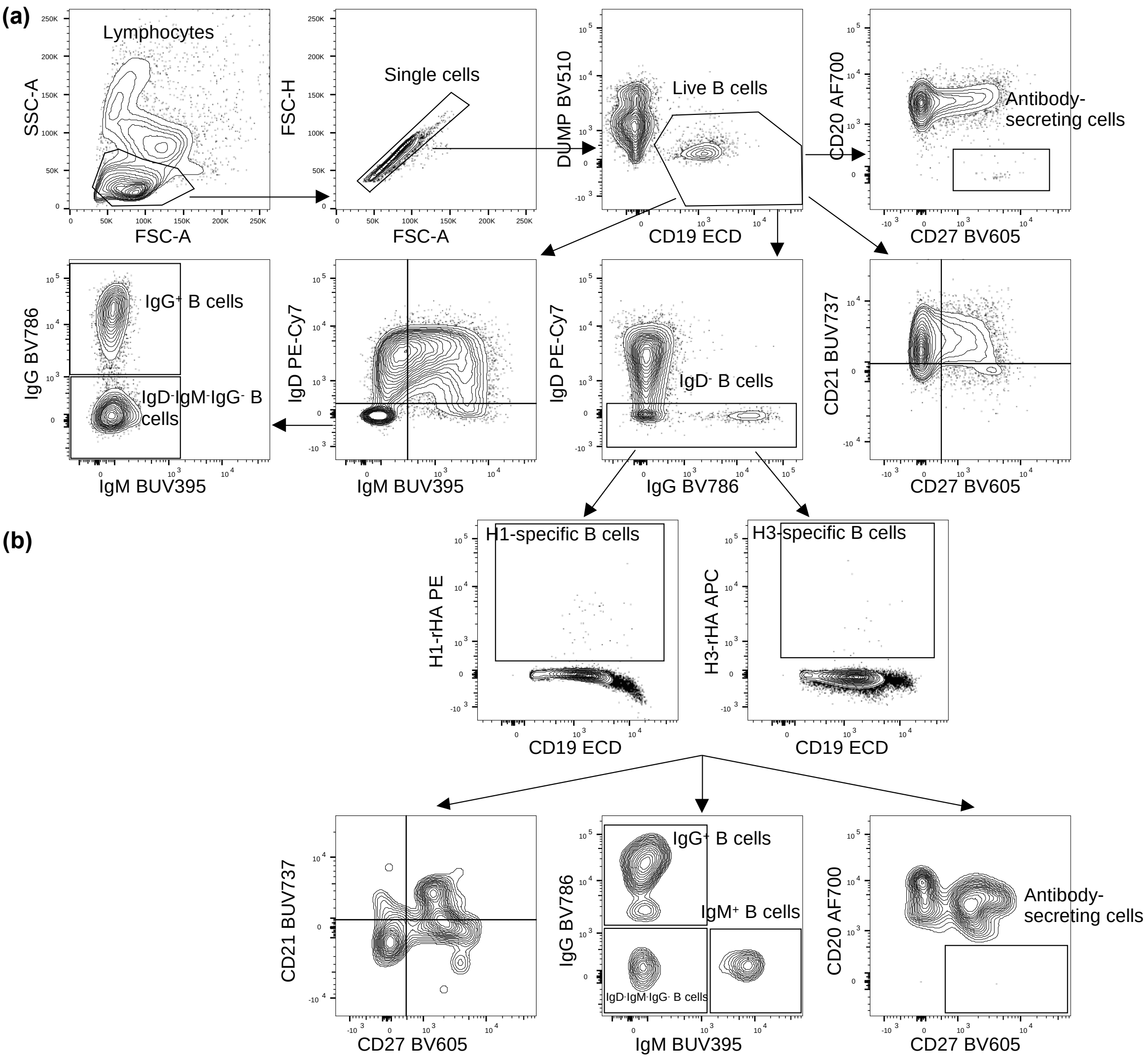

Supplementary figure 3. Gating strategy for B cell subsets and influenza-specific B cells.

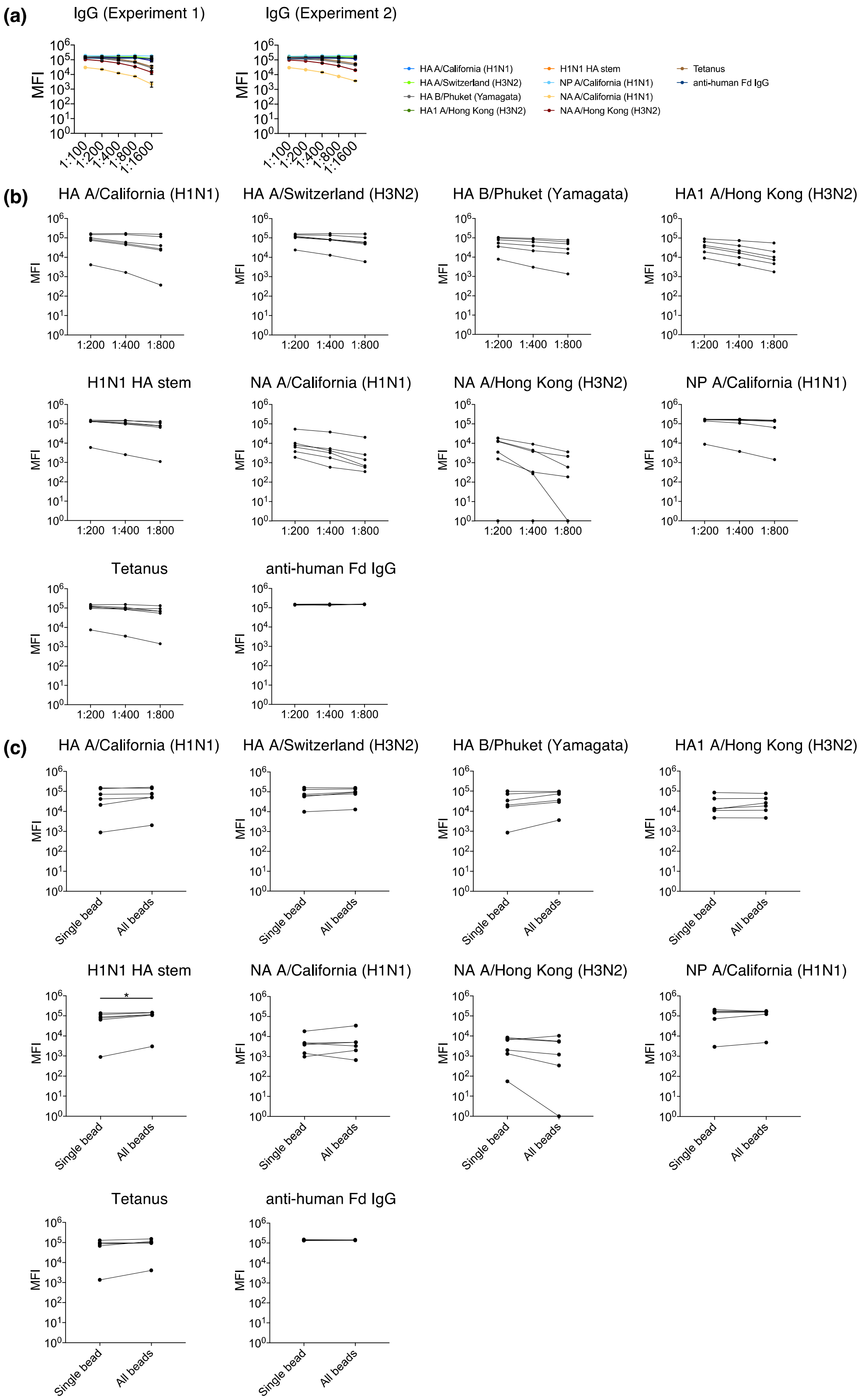

Supplementary figure 4. Optimization of multiplex bead assay conditions. (a) Two-fold serial dilution (1:100 to 1:1600) of a positive sample pool using IgG detector antibody. **(b)** Two-fold serial dilution (1:200 to 1:800) of 6 selected BMT-V cohort serum samples with divergent HAI titres using IgG detector antibody. **(c)** MFI value of six randomly selected BMT-V cohort samples when using single bead or all beads pooled together. Significance was determined using the Wilcoxon test (*$P < 0.05$).

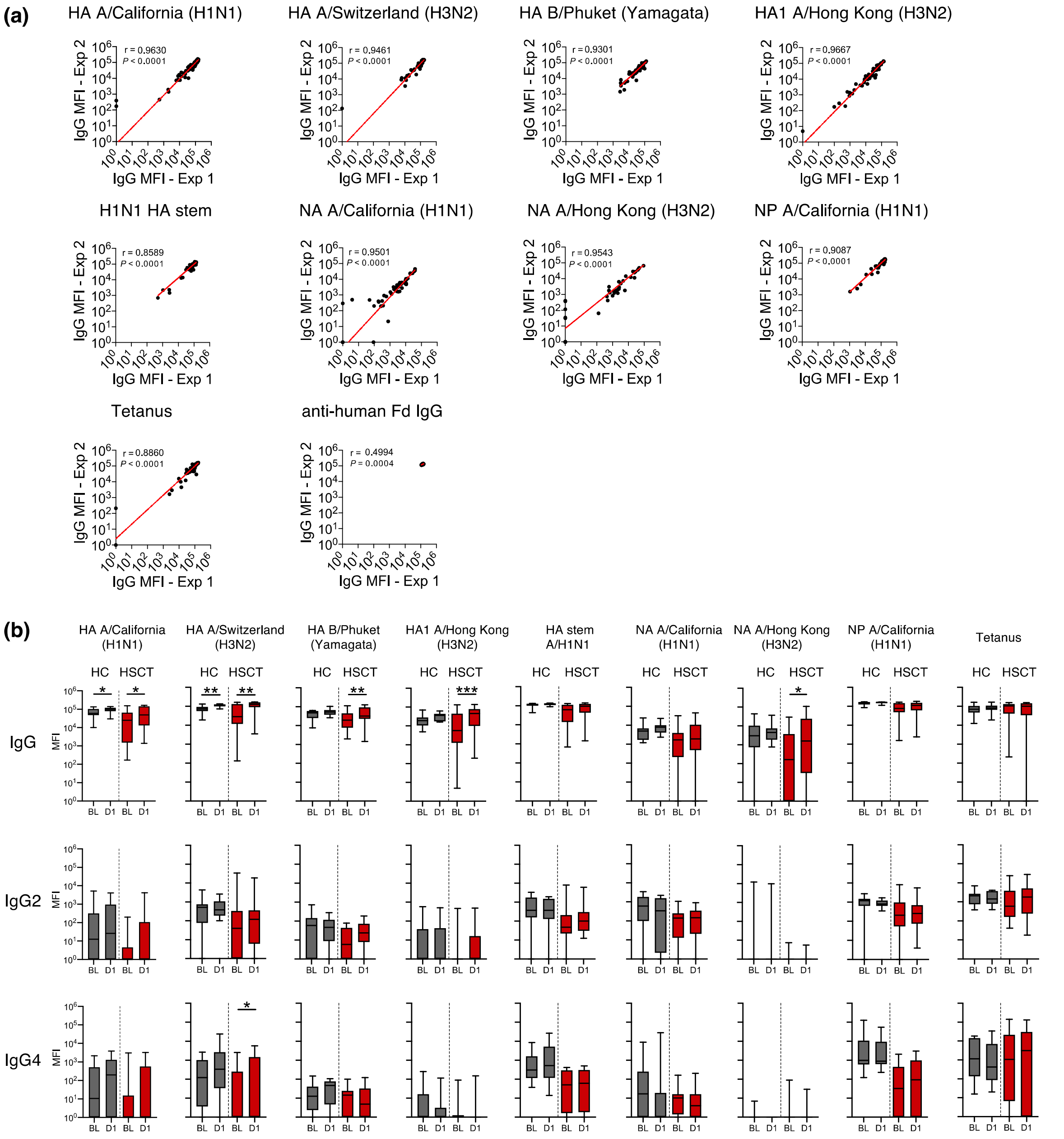

Supplementary figure 5. Reproducibility of the multiplex bead assay. (a) Correlation between the MFI value of the two replicates using IgG detector antibody. Correlation was determined with the Spearman's correlation. **(b)** Level of influenza-specific antibodies for IgG, IgG2 and IgG4 isotypes. Boxes and whiskers indicate median and interquartile range and range respectively. Significance between baseline and after one dose was determined using the Wilcoxon test for each group (n_{HC} = 10, n_{HSCT} = 15; **P* < 0.05, ***P* < 0.01, ****P* < 0.001).
